# Multiclass Classification by Adaptive Network of Dendritic Neurons with Binary Synapses Using Structural Plasticity

**DOI:** 10.3389/fnins.2016.00113

**Published:** 2016-03-31

**Authors:** Shaista Hussain, Arindam Basu

**Affiliations:** School of Electrical and Electronic Engineering, Nanyang Technological UniversitySingapore, Singapore

**Keywords:** active dendrite, structural plasticity, binary synapses, multiclass classification, neuromorphic

## Abstract

The development of power-efficient neuromorphic devices presents the challenge of designing spike pattern classification algorithms which can be implemented on low-precision hardware and can also achieve state-of-the-art performance. In our pursuit of meeting this challenge, we present a pattern classification model which uses a sparse connection matrix and exploits the mechanism of nonlinear dendritic processing to achieve high classification accuracy. A rate-based structural learning rule for multiclass classification is proposed which modifies a connectivity matrix of binary synaptic connections by choosing the best “k” out of “d” inputs to make connections on every dendritic branch (*k* < < *d*). Because learning only modifies connectivity, the model is well suited for implementation in neuromorphic systems using address-event representation (AER). We develop an ensemble method which combines several dendritic classifiers to achieve enhanced generalization over individual classifiers. We have two major findings: (1) Our results demonstrate that an ensemble created with classifiers comprising moderate number of dendrites performs better than both ensembles of perceptrons and of complex dendritic trees. (2) In order to determine the moderate number of dendrites required for a specific classification problem, a two-step solution is proposed. First, an adaptive approach is proposed which scales the relative size of the dendritic trees of neurons for each class. It works by progressively adding dendrites with fixed number of synapses to the network, thereby allocating synaptic resources as per the complexity of the given problem. As a second step, theoretical capacity calculations are used to convert each neuronal dendritic tree to its optimal topology where dendrites of each class are assigned different number of synapses. The performance of the model is evaluated on classification of handwritten digits from the benchmark MNIST dataset and compared with other spike classifiers. We show that our system can achieve classification accuracy within 1 − 2% of other reported spike-based classifiers while using much less synaptic resources (only 7%) compared to that used by other methods. Further, an ensemble classifier created with adaptively learned sizes can attain accuracy of 96.4% which is at par with the best reported performance of spike-based classifiers. Moreover, the proposed method achieves this by using about 20% of the synapses used by other spike algorithms. We also present results of applying our algorithm to classify the MNIST-DVS dataset collected from a real spike-based image sensor and show results comparable to the best reported ones (88.1% accuracy). For VLSI implementations, we show that the reduced synaptic memory can save upto 4X area compared to conventional crossbar topologies. Finally, we also present a biologically realistic spike-based version for calculating the correlations required by the structural learning rule and demonstrate the correspondence between the rate-based and spike-based methods of learning.

## 1. Introduction

There has been significant research in the last decade aimed at designing neuromorphic systems which can emulate the architectural and computational principles of the brain. These systems exploit the spike-based operation of human brain, with minimal power consumption during long inactive periods, to implement power-efficient neuromorphic devices. Moreover, this attempt to mimic the neuronal function can enable us to design event-driven, compact hardware systems which can provide efficient, real-time, intelligent solutions for several applications like robotics and brain-machine interfaces. Conversely, the neuromorphic systems can be used to understand the working principles of brain. The development of event-driven sensors like the artificial retina (Lichtsteiner et al., 2008; Posch et al., 2011; Serrano-Gotarredona and Linares-Barranco, 2013) and cochlea (Liu et al., 2013), which produce continuous and asynchronous spikes encoding the sensory information, make it essential to interface these sensors with spike-based classifier systems to enable the classification of real-world complex stimuli. The spike classification algorithms designed to this effect can also attain large computational power of spiking neural networks (Maass and Schmitt, 1999).

The spike-based neuromorphic systems implemented in very-large-scale integration (VLSI) technology consist of hybrid analog-digital circuits, where the neuronal and synaptic computations are usually performed in analog form on the chip (though TrueNorth Merolla et al., 2014 and Spinnaker Painkras et al., 2013 are notable exceptions) while the synaptic connectivity information is stored on or off-chip in a digital memory. An asynchronous communication protocol called the address-event representation (AER; Boahen, 2000; Choi et al., 2005; Vogelstein et al., 2007; Serrano-Gotarredona et al., 2009), is used to transmit neuronal spikes between neuromorphic chips on a shared fast digital bus. The AER-based neuromorphic systems have the added advantage of reconfigurability since the configuration details of a network are stored in a separate memory, thereby giving the user flexibility to reconfigure the network connectivity.

However, the statistical variations in VLSI devices which reduce the accuracy of the synaptic weights are a major cause for concern in attaining performance comparable to software simulations. To mitigate the effect of increasing device mismatch with progressively shrinking transistor sizes, the usual solutions are to increase device sizes or employ a large number of neurons, both of which increase chip area. For example, a spiking network classifier implemented on a neuromorphic hardware system achieved performance comparable with standard machine learning linear classifier and exhibited tolerance against variability by using population coding (Schmuker et al., 2014). However, a limitation of this model is the large number of high resolution weights used to attain the reported performance. Similarly, spike classifiers consisting of Restricted Boltzmann Machine (RBM) constructed with integrate and fire neurons, use a large number of recurrent synaptic connections (O'Connor et al., 2013; Neftci et al., 2014) rendering these algorithms impractical for compact VLSI implementation. A simple and robust solution to the problem of device mismatch can be obtained by using binary synaptic weights. A spike-based STDP learning rule using bistable synapses was implemented in Brader et al. (2007) with the VLSI implementation in Mitra et al. (2009) to classify complex stimuli. A pool of neurons was used to improve the classification accuracy by employing a voting scheme, which again leads to the problem of increased number of synapses. Digital implementations do not suffer from mismatch issues like their analog counterparts; however, the usage of a lot of memory to implement high resolution weights for deep networks increases chip area significantly.

A spike classification model proposed in Hussain et al. (2013, 2015) offers a solution to the problem of large number of synaptic weights by using a structural plasticity based learning rule which involves formation of *sparse* connections with *binary* weights. Moreover, it was shown that a simple correlation-based learning rule provides an alternative to the traditional weight-based learning rules and is more suitable for implementation on neuromorphic chips. The problem of reduced memory capacity of a network with binary synapses as compared with that of continuous-valued synaptic weights (Senn and Fusi, 2005) is alleviated by the use of nonlinear dendritic processing, which emerges due to the presence of voltage gated ion channels (Magee, 2000; London and Hausser, 2005). A limitation of this method is that it uses a preassigned network size and the number of dendrites and synapses required for solving a classification problem of given complexity is not known. Hence, it is desirable to use an approach which learns to allocate the required number of synaptic resources for a specific problem.

Several adaptive approaches have been used to control the network size and structure. A constructive approach involves training with a minimal architecture, for example a single hidden-layered network with one hidden neuron, and then adding further hidden units and weights to implement the desired mapping (Kwok and Yeung, 1997; Lahnajarvi et al., 2002; Islam et al., 2009). The second approach for automating the design of appropriate neural network is by pruning in which a network larger than necessary is trained and then redundant connections and/or neurons are removed until an acceptable solution is obtained. A group of pruning algorithms eliminate a neuron or a connection which have the least effect on the error function (Karnin, 1990). The other group of pruning algorithms are referred to as regularization methods which add a penalty term proportional to the sum of weights to the objective function. Hence, the unnecessary weights are driven to zero during training and are eliminated in effect (Kwok and Yeung, 1996). Several other pruning methods are reviewed in Islam et al. (2009) and Reed (1993). Another class of algorithms that are developed to control the network size and structure use a hybrid approach of combining the constructive and pruning methods (Fiesler, 1994). The growth and pruning algorithms discussed here have not considered the number of dendrites as an adaptive parameter.

In this paper, we present a multiclass classifier using neurons with nonlinear dendrites (NNLD) and a structural learning rule for finding sparse, binary weight matrices. The unique contributions of this work are: (1) Showing that for ensembles of NNLD with the same number of synapses, having a single dendritic branch (perceptron) or having too many dendrites are sub-optimal; the optimal cases is a moderate size of the dendritic tree. (2) Developing an algorithm that adapts the size of the dendritic tree for each class according to the difficulty of classifying that pattern category. (3) Applying this network to the problem of handwritten digit recognition task from MNIST and MNIST-DVS datasets to show its benefit in achieving high accuracy with very small memory usage for weights. (4) Demonstrating memory size reductions possible in VLSI implementations by using this training method.

The paper is organized as follows. First, the rate-based multiclass spike pattern classification model is presented in Section 2.1.1, followed by the description of an ensemble method which combines the outputs of several dendritic classifiers to obtain improved classification accuracy in Section 2.1.2. In Section 2.1.3, we propose an adaptive structural learning scheme which involves growth of the network by adding dendrites based on the progress of learning process. The performance of different learning schemes on classification of handwritten digit samples is demonstrated in Sections 3.2–3.4, while noise sensitivity analysis is in Section 3.5 and analysis of dendrite weights in Section 3.6. Next, in Section 3.7, we present an approach to optimize the performance of our method by utilizing theoretically determined optimal neuron topology. Finally, the evaluation of our algorithm on event-based MNIST-DVS dataset is presented in Section 3.8 followed by a comparison of the performance of our model with the results obtained using other spike-based classification algorithms in Section 3.9. The relevance of our work in terms of the biological plausibility, comparisons with other related studies, hardware considerations and a discussion of future work is included in Section 4.

## 2. Materials and methods

### 2.1. Spike classification with nonlinear dendrites and structural learning

**Figure 1:**
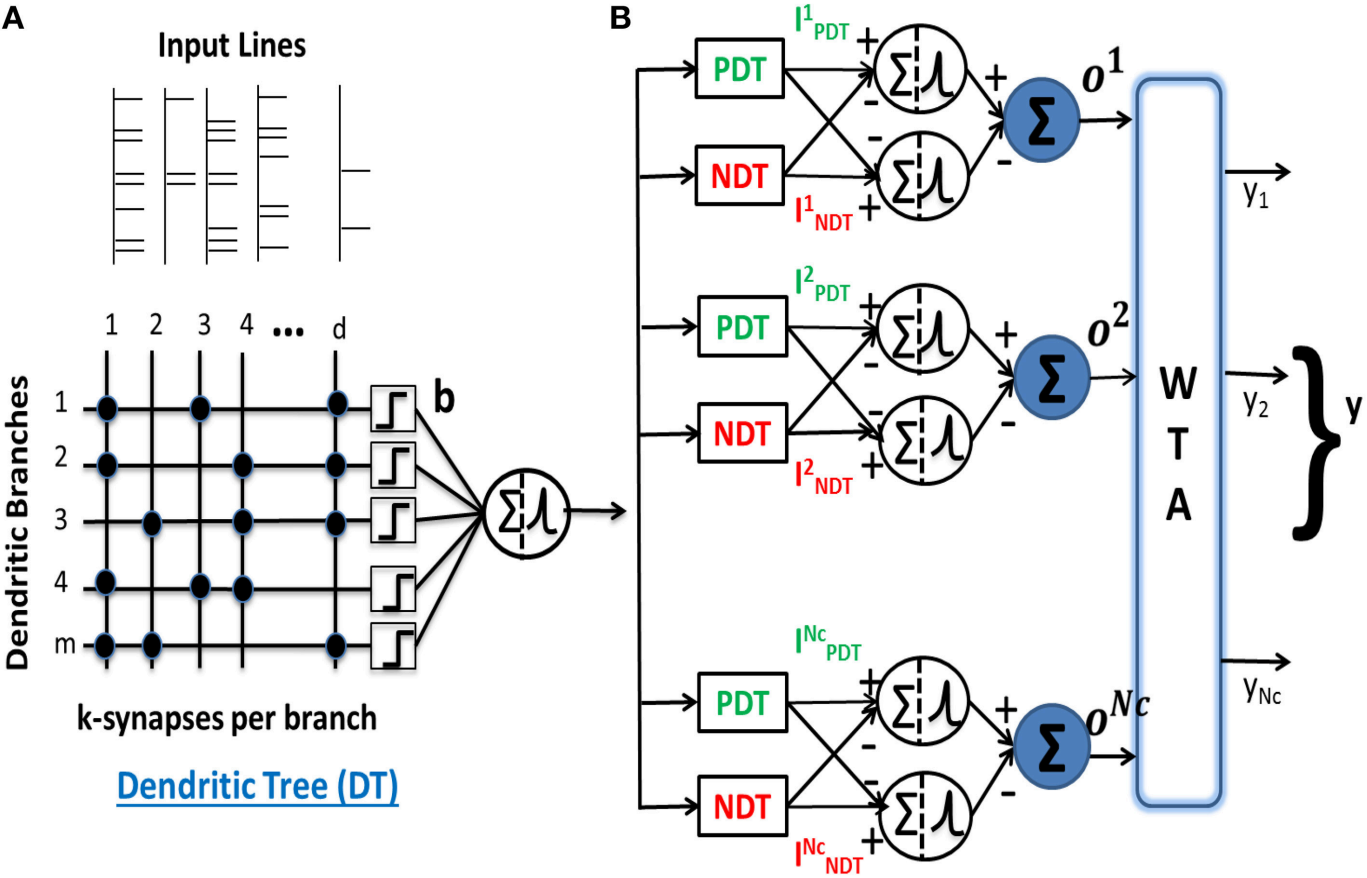
**(A)** A neuron consisting of nonlinear dendrites characterized by the lumped nonlinear function *b*() for every dendritic branch. It forms *k* binary connections from *d* input afferents on *m* dendrites. **(B)** Architecture of a multiclass pattern classifier consisting of nonlinear neurons receiving inputs from PDT and NDT and the neurons connected to a WTA to compare the outputs of the *N*_*C*_ classes. The output of the classifier is equal to 1 for the class corresponding to the highest output *o*^μ^.

We have proposed a margin-based neuron model with nonlinear dendrites (NLD) for spike pattern classification (Hussain et al., 2015). The model comprises nonlinear neurons having lumped dendritic nonlinearity where a nonlinear neuron (NL-neuron) consists of multiple (*m*) dendritic branches with each branch governed by its nonlinearity *b*() and *k* excitatory synapses on each branch driven by one of the *d* input components (Figure 1A). The model uses binary weight for the *i*th synapse on the *j*th dendrite, *w*_*ij*_ ∈ {0, 1}, where *i* ∈ {1, ⋯*d*}, *j* ∈ {1, ⋯*m*}. The advantage of such binary weight connections are in hardware implementations since they require much less memory resources, are more resistant to mismatch in analog implementations and also enable easy digital implementations as done in Merolla et al. (2014). Moreover, we enforce sparse connectivity on each dendritic branch (*k* < < *d*) and allow the learning to choose the best “k” connections on each branch. Hence, we can write:

(1) $$\sum_{i=1}^{d}w_{ij}=k\text{ for }j=1,\cdots ,m$$

This model was used to perform supervised binary classification of spike patterns. In our current work, we extend the model to perform multiclass classification of spike patterns belonging to *N*_*C*_ classes. The multiclass classifier consists of (+) and (−) neurons corresponding to all *N*_*C*_ classes. The inputs to the neurons for class μ are received from a pair of excitatory and inhibitory dendritic trees, also referred to as positive and negative dendritic trees (PDT and NDT), respectively, each with *m* dendrites. These input currents for class μ neurons are given by:

(2) $$I_{in}^{\mu +}(t)=I_{PDT}^{\mu }(t)-I_{NDT}^{\mu }(t)$$

(3) $$I_{in}^{\mu -}(t)=I_{NDT}^{\mu }(t)-I_{PDT}^{\mu }(t)$$

where μ ∈ {1, ⋯*N*_*C*_} and $I_{PDT}^{\mu }\left(t\right)$, $I_{NDT}^{\mu }\left(t\right)$ are the currents generated by the PDT and NDT of class μ, calculated by taking the sum of dendritic output currents $I_{PD,j}^{\mu }\left(t\right)$ and $I_{ND,j}^{\mu }\left(t\right)$ respectively, which can be expressed as nonlinear functions of the total synaptic current on a dendrite. To make notations simpler for the ease of reading, we drop the superscript μ for the μth class in the rest of the paper except where the outputs of two or more classes are compared. Hence, we denote the PDT/NDT currents for class μ neuron as *I*_*DT*_(*t*) and the output current of the *j*th dendrite of PDT/NDT as *I*_*D, j*_(*t*). Therefore, the equations for *I*_*DT*_(*t*) can be written as:

(4) $$I_{DT}(t)=\sum_{j}I_{D,j}\left(t\right)$$

(5) $$=\sum_{j}b\left(\sum_{i}w_{ij}\;\left(\sum_{t_{ij}<t}K(t-t_{ij})\right)\right)$$

(6) $$=\sum_{j}b(z_{j}(t))$$

Here *K*(*t*) denotes the postsynaptic current (PSC) generated by the spike input at a synapse of the PDT or NDT of class μ and is given by Gutig and Sompolinsky (2006):

(7) $$K(t-t_{ij})=I_{0}\left(exp[-(t-t_{ij})/\tau_{f}]-exp[-(t-t_{ij})/\tau_{r}]\right)$$

where *t_ij_* denotes the times at which spikes arrive at this synapse; *I*_0_ is the normalization constant; and τ_*f*_ and τ_*r*_ are the fall and rise time constants of the PSC respectively; *z_j_*(*t*) is the total synaptic activation on the *j*th dendrite and *b*(*z_j_*(*t*)) is the dendritic nonlinear function given by:

(8) $$b(z_{j}(t))=g(z_{j}(t)-z_{leak,j}(t)){\left(z_{j}(t)-z_{leak,j}(t)\right)}^{2}$$

Here, *g*() is a Heaviside step function that gives an output 1 at all times where the argument (*z_j_*(*t*) − *z_leak,j_*(*t*)) is positive and 0 otherwise and *z_leak,j_*(*t*) is the average synaptic activation on the *j*th branch corresponding to the initial random connections. The use of *z_leak,j_*(*t*) term serves to balance the excitation on each branch by subtracting the mean activation level on that branch, which can be regarded as a signal from a pool of inhibitory neurons. Finally, the spike output *n_spk_* of a neuron of class μ, receiving the input current *I_in_*(*t*), is generated using the leaky integrate and fire neuron model described next.

The dynamics of the membrane potential *V*_*m*_(*t*) of the (+) and (−) neurons of class μ is explained through the equations given below:

(9) $$\tau_{V}\frac{dV_{m}}{dt}=(u-V_{m})+I_{in}(t)$$

(10) $$\begin{array}{l} \;\tau_{u}\frac{du}{dt}=-u\\ \text{If }V_{m}\geq V_{thr},V_{m}\to V_{reset};\\ \;u\to u_{reset}\\ \;u_{reset}=V_{reset}<0\\ \;n_{spk}\to n_{spk}+1 \end{array}$$

where *u* denotes a hyperpolarization variable which relaxes back to 0 with a time constant τ_*u*_ and is set to *u*_*reset*_ after a postsynaptic spike and τ_*V*_ and τ_*u*_ are the time constants governing the fast and slow dynamics of the membrane voltage and hyperpolarization respectively. Here, the variables *V*_*m*_(*t*), *u* and *n*_*spk*_ are used to describe the dynamics of both (+) and (−) neurons of class μ and the input current *I*_*in*_(*t*) can be computed using Equations (2) and (3).

As shown in Figure 1B, the outputs of all the (+) and (−) neurons are connected to a WTA circuit to generate the overall classifier output as:

(11) yμ=g(ospkμ−ospkν),ospkν≥ospkξ,∀ν,ξ=μ

where μ, ν, ξ are integers in the range [1, *N*_*C*_] and *y*_μ_ is the binary-valued μ^*th*^ component of the *N*_*C*_-dimensional output *y*, $o_{spk}^{\mu }=n_{spk}^{\mu +}-n_{spk}^{\mu -}$; $n_{spk}^{\mu +}$ and $n_{spk}^{\mu -}$ are the spike counts of the (+) and (−) neurons of class μ respectively.

In this paper, we have developed rate-based and spike-time-based learning schemes for our multiclass classification model with NLDs. For these schemes using binary synapses, we present structural learning rules, which involve the modification of connection matrix instead of connection weights. The motivation for using rate-based learning is driven by the fact that the spike-time-based learning requires significant processing time and therefore, to mitigate this problem we have used the strategy of training on mean rate inputs to reduce the training time. With the use of faster rate-based learning, we further propose an ensemble method which combines the outputs of several NLD classifiers in order to achieve performance gain over individual classifiers. We also develop an adaptive learning scheme which learns to allocate the required number of dendrites to a neural network along with learning the connection patterns on these dendrites. The bio-realistic spike-based approximation of the structural learning rule for multiclass classification is an extension of an online spike-based binary classification rule proposed in our earlier work (Hussain et al., 2015). The derivation of this rule and the correspondence between our spike-based and rate-based learning schemes are presented in the Appendix. Next, we describe the rate-based learning rule.

#### 2.1.1. Rate-based learning scheme for multiclass classification

The rate-based learning rule for NLD model is valid for rate encoded inputs like Poisson spike trains and place/synchrony encoded single spike patterns. This validity results from the fact that for such spike inputs, the average synaptic activation $z_{syn,ij}=\frac{1}{T}\int_{0}^{T}\sum_{t_{ij}}K(t-t_{ij})dt$ is directly proportional to the input arriving at that synapse, *x*_*ij*_, where *T* is the pattern duration (Hussain et al., 2015). This reduced model was developed to improve the training time and it uses encoded binary vectors obtained by mapping spike train with “high” firing rate or single spike to binary value “1” and spike train with “low” firing rate or absence of spike to binary value “0.” A rate-based NLD model for multiclass classification was proposed in Hussain et al. (2014), which consisted of *N*_*C*_ neurons representing *N*_*C*_ classes. Here, we present a modified version of this model consisting of PDT and NDT corresponding to each of the *N*_*C*_ classes. The output of the PDT and NDT of class μ to input *x* is given by:

(12) $$a_{PDT}(x)=\sum_{j=1}^{m}b\left(\sum_{i=1}^{d}w_{PDT,ij}x_{PDT,ij}\right)$$

(13) $$a_{NDT}(x)=\sum_{j=1}^{m}b\left(\sum_{i=1}^{d}w_{NDT,ij}x_{NDT,ij}\right)$$

The difference of outputs of the positive and negative dendritic trees of each class, *o*(*x*) = (*a*_*PDT*_(*x*) − *a*_*NDT*_(*x*)), are then used as inputs to the WTA circuit which computes the overall output and therefore decision of the classification task, *y*(*x*) using a similar logic as in Equation (11).

(14) yμ(x)=g(oμ(x)−oν(x)),oν(x)≥oξ(x),∀ν,ξ=μ

During training, the classifier output is computed using a margin-based output function, *g*_*margin*_(*x*) instead of *g*(*x*) to enforce a margin around the classification boundary. The *g*(*x*) function is only used during the testing phase to calculate the output. Therefore, the classifier output during training calculated using the *g*_*margin*_(*x*) function is given by:

(15) yμ(x)=gmargin(oμ(x)−oν(x)),oν(x)≥oξ(x)yν(x)=gmargin(oν(x)−oμ(x)),∀ν,ξ=μ

The function *g*_*margin*_() is defined as:

(16) $$\begin{array}{l} g_{margin}(\alpha )=1\text{ if }\alpha \geq \delta \\ \;=0\text{ if }\alpha \leq -\delta \\ \;=\frac{0.5}{\delta }\alpha +0.5\text{ otherwise} \end{array}$$

where the margin is set using different values of the parameter δ for different classes to which input patterns belong. The value of margin δ for each class is determined using the following steps:

1. Multi-class model is first trained using the *g*(*x*) function, where the output of the model is calculated using 12. Connection matrices for all neurons are saved.
2. Cross validation set patterns are presented and the values α_μν_ = (o^ν^(*x*) − o^μ^ (*x*)) are recorded for all the cases for which patterns from class μ are misclassified as belonging to class ν.
3. The set of α_μν_ values for each class μ ∈ {1, ⋯*N*_*C*_} are saved. The margin for class μ, δ^μ^ is set to the highest value of α_μν_.
4. δ^μ^ value is reduced to 80% of its present value whenever learning algorithm gets stuck in the same local minimum for 5 consecutive times.

The learning process which is based on the mechanism of structural plasticity involves formation and elimination of synaptic connections. The connections are modified by computing a metric based on correlation values *c*_*ij*_ for each synapse of the PDT and NDT. Hence, the learning rule for the rate-based learning scheme is given by:

(17) $$\begin{array}{l} \text{For class }\mu ,\text{ for PDT}:\\ \;c_{ij}^{PDT}=<x_{ij}^{PDT}b_{j}^{PDT}sgn(y_{\mu }^{d}-y_{\mu })> \end{array}$$

(18) $$\begin{array}{l} \text{For class }\mu ,\text{ for NDT}:\\ \;c_{ij}^{NDT}=-<x_{ij}^{NDT}b_{j}^{NDT}sgn(y_{\mu }^{d}-y_{\mu })> \end{array}$$

where the desired output *y*^*d*^ is available as the teacher signal during the training phase and is a *N*_*C*_-dimensional binary vector consisting of (*N*_*C*_ − 1) zeros and a 1 corresponding to the class to which the input pattern belongs; *b_j_* is the output of the *j^th^* dendrite of a PDT or NDT; *sgn*() is the signum function with a value of 1 for $y_{\mu }^{d}>y_{\mu }$, −1 for $y_{\mu }^{d}<y_{\mu }$ and 0 for $y_{\mu }^{d}=y_{\mu }$; and the output *y* is computed using Equation (15).

The connection changes are done by using the following logic: a synapse with the smallest *c*_*ij*_ value corresponds to a poorly-performing synapse and is a candidate for replacement. To replace this synapse, a set of silent synapses are first formed on the chosen branch as possible candidates for the new connection. The silent synapse with the highest *c*_*ij*_ is chosen as the replacement. At the start of the learning process, the input connections for all the PDT and NDT corresponding to *N*_*C*_ classes each with *m* dendritic branches, *k* synaptic contacts per branch and *s* = *m* × *k* total synapses are initialized by randomly selecting afferents from among *d* input lines with weight *w*_*ij*_ = 1. Training set consisting of *P* input patterns (*x*) belonging to *N*_*C*_ classes is presented. The learning process comprises the following steps in every iteration consisting of the presentation of all *P* patterns:

1. The misclassification error rate is calculated by taking the average of the fraction of patterns for which $y_{\mu }\neq y_{\mu }^{d}$ for any μ ∈ {1, ⋯*N*_*C*_}.
2. For each dendritic tree of class μ, a random set *T* consisting of *n*_*T*_ (< *s*) synapses is selected as candidates for replacement.
3. The synapse with the lowest *c*_*ij*_ in *T* is targeted for replacement and the dendrite *j*_*T*_ on which it is located is identified. A random replacement set *R* is created by placing *n*_*R*_ “silent” synapses from *d* input lines (*n*_*R*_ < *d*) on the $j_{T}^{th}$ dendrite. The synapse with the lowest *c*_*ij*_ in *T* is replaced by the best-performing (maximum *c*_*ij*_) synapse in *R*. The silent synapses do not contribute to the calculation in step (1).
4. Synaptic connections are modified if the replacement led to either a reduction or no change in error rate. If the error increased with the replacement, a new replacement set *R* is created. If the error does not decrease after repeating this step *n*_*ch*_ (= 50) times, we assume a local minimum is encountered. We then do a replacement with the last choice of *R*, even if it increases error in an attempt to escape the local minimum. The connection matrix corresponding to the local minimum that gave the least error is saved.
5. Steps (1)–(4) are repeated until either all the patterns have been memorized or *n*_*min*_ (= 150) local minima are encountered. At this point, the learning process is stopped. The connection patterns corresponding to the best minimum become the final learned synaptic connections of the neuron.

After the completion of training, the final learned connections are saved and then used to calculate the error rate on the spike test pattern set for the testing phase. This is done by mapping the learned connections onto an equivalent spiking network by introducing synaptic integration and spike initiation mechanisms. The testing classification output is computed using Equation (11).

#### 2.1.2. Ensemble learning scheme for multiclass classification

**Figure 2:**
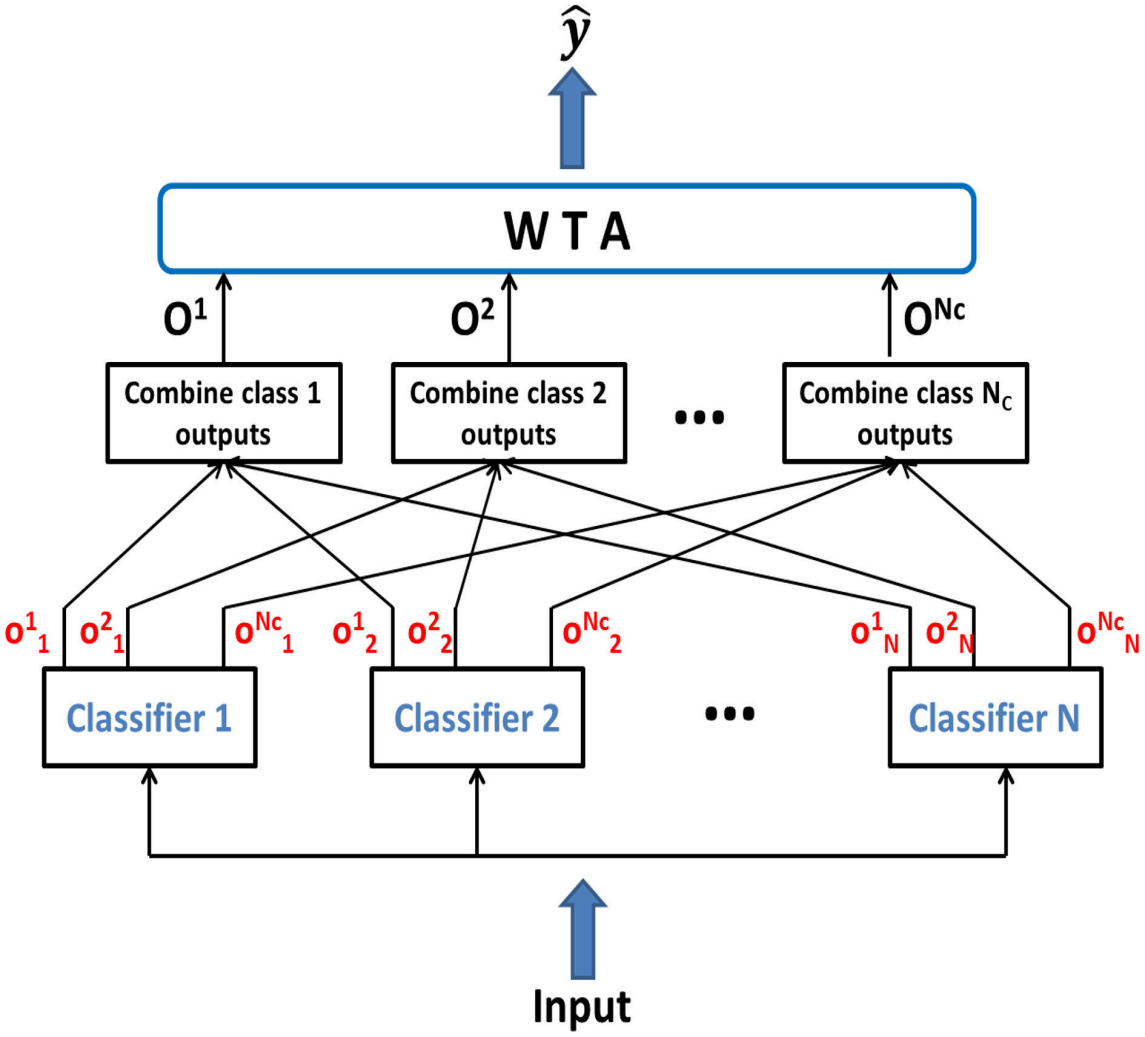
**Ensemble classifier combining the intermediate class-specific outputs $o_{n}^{\mu }$ to compute the combined class outputs *O*^μ^, which are compared by the WTA to generate the final classifier output $\hat{\text{y}}$**.

We have also used an ensemble method in this work, where several classifiers when combined together yield better classification accuracy than any of the single classifiers in the ensemble. Our ensemble model consists of individually trained NLD classifiers which are then combined to classify novel test patterns. Since, previous research has demonstrated that combining identical classifiers doesn't produce any gain over individual classifiers (Opitz and Maclin, 1999), hence we created ensemble by combining several classifiers which are individually trained and disagree on their predictions. The complementary information about the novel patterns obtained from different classifiers can be exploited to produce a more accurate overall output. In order to generate different predictions for different classifiers, individual networks were initialized with different random synaptic connections. Figure 2 shows the basic framework for the classifier ensemble scheme. It consists of *N* individually trained multiclass classifiers as members of the ensemble. Each classifier generates the intermediate output, $o_{n}^{\mu }(x)$, which is the difference in outputs of PDT and NDT for class μ of the *n*^*th*^ classifier. The intermediate outputs are combined in a class-specific manner to give $O^{\mu }(x)=\sum_{n=1}^{N}o_{n}^{\mu }(x)$. Finally, the ensemble output is generated using:

(19) y^μ(x)=g(Oμ(x)−Oν(x)),Oν(x)≥Oξ(x),∀ν,ξ=μ

The performance of ensemble model gives us an insight into the number of component classifiers needed in an ensemble. However, it is impractical to use a large number of classifiers in an ensemble due to the long training time required and also because it leads to increased synaptic resources. Therefore, we use the ensemble created with a few classifiers as a trade-off between trainability and accuracy. Moreover, it is not clear what level of complexity is required for individual classifiers and whether an ensemble of perceptrons will perform better than that of complex dendritic trees. To address this problem, we have developed an adaptive learning rule for allocating the number of dendrites according to the difficulty of the problem being solved as well as the difficulty to learn a specific class. This scheme is discussed next.

#### 2.1.3. Adaptive learning scheme for multiclass classification

For the learning schemes described above, a fixed number of dendrites (*m*) was chosen and assigned to all the dendritic trees in the model. Since, it is difficult to choose an optimal value of *m* that matches the complexity of a given problem, we have proposed an algorithm in which the number of dendrites corresponding to each class is learned during the training process. The value of *m* is adapted for each class dendritic trees independently while the number of synapses per branch, *k*, is kept constant throughout. Hence, the learning process generates the relative sizes of the connection matrix for each neuron as well as the optimal sparse connections within that matrix. The learning rule used is same as that for the rate-based scheme with fixed *m* except that all the computations in every iteration are done for the current value of *m* for each neuron. Additional steps are included in the learning algorithm to adapt the value of *m*. After making a correlation-based connection change, we check if the learning process for a particular class μ has slowed down using the following steps:

1. If the error rate for class μ, which is calculated as the average of the number of patterns for which $y_{\mu }\neq y_{\mu }^{d}$, does not decrease after *n*_*ch*_ (= 50) iterations, the learning process for class μ has encountered a local minimum. In this case, a new dendrite is added to both PDT and NDT corresponding to class μ, if the error is the highest amongst all the *N*_*C*_ classes.
2. This is done by drawing *k* random connections from the *d* input lines to represent a new dendrite. The connection matrix is updated by appending the new dendrite to the existing matrix as a new row. The value of *m* for class μ is incremented according to:
  (20) $$\begin{array}{l} m^{PDT}\to m^{PDT}+>1\\ m^{NDT}\to m^{NDT}+1 \end{array}$$
3. After adding a dendrite, error rate for cross validation set is computed. If this validation error increases in the last 3 dendrite addition steps, the learning process is stopped. The connections are frozen and saved for the testing phase.

The above steps are added after the step (4) of the rate-based learning algorithm in Section 2.1.1 as steps 4.1, 4.2, and 4.3. All the steps from (1)–(4.3) are then repeated until learning stops by one of the stopping conditions discussed in step (5).

**Figure 3:**
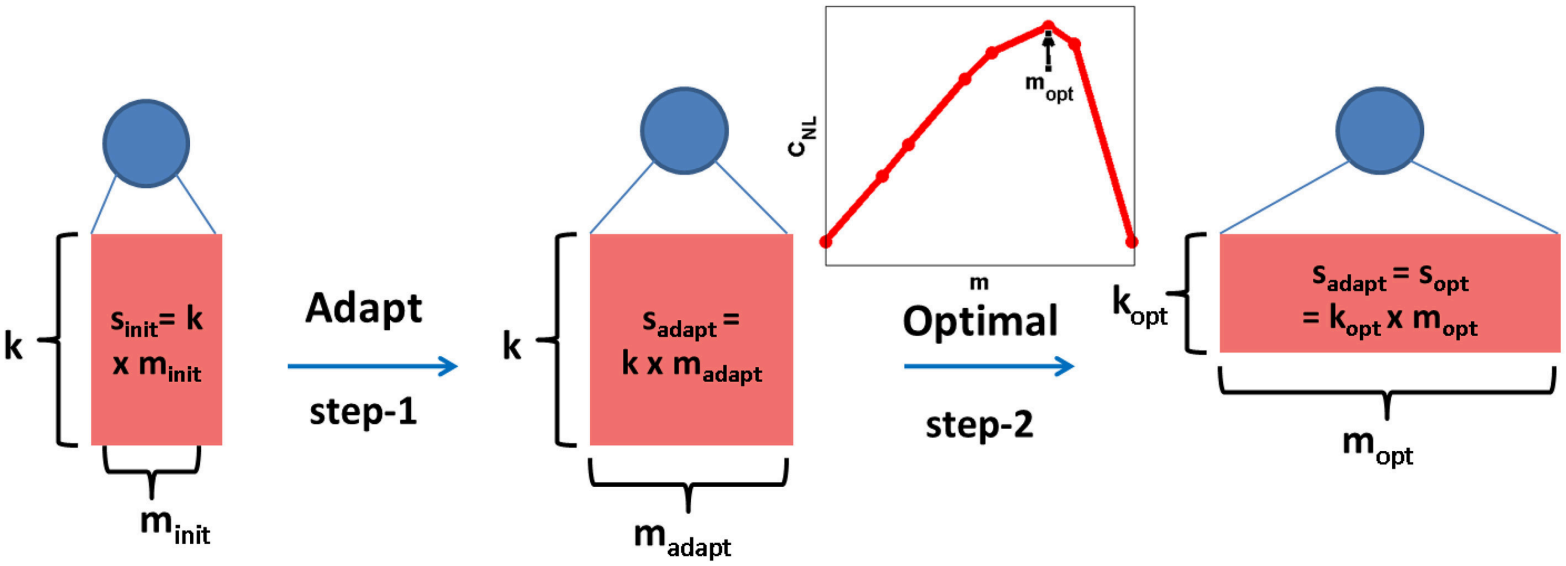
**Dendritic classifier is trained adaptively by adding dendrites (step-1) and keeping the number of synapses/dendrite (*k*) as a constant for all neurons**. The total number of synapses for the μ*th* neuron learned in this manner ($s_{adapt}^{\mu }$) is fixed and the corresponding optimal topology ($m_{opt}^{\mu }$, $k_{opt}^{\mu }$ inset plot) is theoretically determined (step-2). The length of sides of the rectangle denote *m* and *k* values and the area represents the total number of synapses, *s*.

This adaptive learning method is guided by the level of difficulty of each neuron's classification task and moreover, it is used to learn the relative size of each neuron and not the optimal neuron topology. While performing this adaptation, we keep the number of synapses per branch, *k*, to be a constant for all neurons and just vary the number of dendrites per neuron, *m*. Hence, at the end of the adaptation, both PDT and NDT of the μth class have a total number of synapses $s_{adapt}^{\mu }$ given by $s_{adapt}^{\mu }=m_{adapt}^{\mu }\times k$, where $m_{adapt}^{\mu }$ is the number of dendritic branches learned for the μth class neurons after adaptation. This is shown as the step-1 in Figure 3, where the topology of a class neuron is denoted by the rectangle with its sides representing *m* and *k* values.

However, from theoretical considerations of function counting as shown in Poirazi and Mel (2001), the theoretical capacity of a neuron with nonlinear dendrites with a fixed value of total number of synapses (*s*_*opt*_) is maximum for a relatively large value of *m*_*opt*_ and small value of *k*_*opt*_. This theoretical capacity was given by the combinatorial expression derived by counting all possible ways in which *d* afferents can connect to synapses on dendrites resulting in distinct memory fields (Poirazi and Mel, 2001). Hence, for a neuron with NLDs, the capacity *C*_*NL*_ in bits was calculated as:

(21) $$C_{NL}=2log_{2}\left(\begin{array}{c} f+m-1\\ m \end{array}\right)$$

where $f=\left(\begin{array}{c} k+d-1\\ k \end{array}\right)$ is the number of distinct branch functions. As shown in the inset plot for the optimal topology determination step in Figure 3, *C*_*NL*_ plotted as a function of *m* has a maximum at *m*_*opt*_ for a fixed value of *s*_*opt*_. These derivations were also discussed in our previous work (Hussain et al., 2015). We can now use this theory to change the structure of the μth neuron to optimize capacity while preserving the same number of synapses. Hence, by setting $s_{opt}^{\mu }=s_{adapt}^{\mu }$ (fixing the area of rectangle as shown in step-2 in Figure 3), the theoretical optimal topology ($m_{opt}^{\mu }$, $k_{opt}^{\mu }$) is determined such that $s_{opt}^{\mu }=s_{adapt}^{\mu }\;=\;m_{opt}^{\mu }\times k_{opt}^{\mu }$. The use of this approach to boost the capacity of our adaptively learned dendritic network is demonstrated in Section 3.7.

## 3. Results

### 3.1. Input generation

We have demonstrated the performance of our multiclass classification NLD model on the MNIST dataset consisting of grayscale images of handwritten digits belonging to one of the *N*_*C*_ = 10 classes (0 to 9). It is a benchmark machine learning dataset used previously to test the performance of many classification algorithms (LeCun et al., 1998). The input patterns consisting of 28 × 28 images were converted into *d* = 784 dimensional binary vectors by thresholding. The training set consists of 20, 000 patterns randomly selected from the full MNIST dataset, with equal number of samples of each digit. The testing set consists of a total of 10, 000 patterns. In case of adaptive learning scheme, 20% of the training patterns comprise the cross validation set which is used to compute the error whenever a dendrite is added to the network and finally to stop adding dendrites when the cross validation error doesn't reduce further. The learning procedure is stopped when either all the patterns are correctly classified or when 150 minima are encountered. For different training runs 150 minima are encountered in different number of learning iterations and for each data point generated, this training process is repeated 3 times.

The binary input vectors are used for rate-based training. For spike-based training, patterns of single spikes are used, which are generated by mapping binary input “*x*_*i*_ = 1” to a single spike arriving at *T*_*syn*_ = 100 ms and “*x*_*i*_ = 0” to no spike, where the stimulus duration *T* = 200 ms. Testing for both forms of rate-based and spike-based learning is done on two types of spike inputs: 1) place/synchrony code of single spikes with jittered spikes arriving within a time window $\left[T_{syn}-\frac{\Delta }{2},T_{syn}+\frac{\Delta }{2}\right]$ corresponding to binary input “1” and no spike for “0” input and different amounts of jitter Δ; and 2) rate encoded Poisson spike trains with mean firing rates of *f*_*high*_ and *f*_*low*_ mapped to binary inputs “1” and “0” respectively. The Poisson spike inputs have only been used to test the noise sensitivity of the model. The parameters used for the rate-based and spike-based models are given in Tables 1, 2 respectively, unless stated otherwise.

**Table 1:** **Parameters for rate-based multiclass model**.

****d****	****m****	****k****	****n**_**R**_**	****n**_**T**_**	****T**_**syn**_**
784	10	10	25	25	100 ms

**Table 2:** **Parameters for spike-based multiclass model**.

**τ_*V*_**	**τ_*u*_**	**τ_*pre*_**	**τ_*post*_**	***V*_*thr*_**	***T***	***T*_*syn*_**
5 ms	200 ms	10 ms	200 ms	0.1 mV	200 ms	100 ms

The significance of using these spike inputs can be understood by considering that single spike representations are commonly used in time-to-first-spike (TTFS) imagers (Qi et al., 2004; Chen and Bermak, 2007). These imagers when presented with binary images such as in the MNIST dataset used in this work, generate a cluster of spikes corresponding to the white pixels and another cluster much later in time corresponding to the black pixels. Such place/synchrony code is also abundantly used in neuroscience (Gerstner and Kistler, 2002). Moreover, rate encoded outputs are commonly available from neuromorphic sensors such as the artificial cochlea in Chan et al. (2007). Such rate encoded Poisson spike trains are also often used to test the performance of neuromorphic classifiers as demonstrated in Marti et al. (2015), O'Connor et al. (2013), and Brader et al. (2007).

### 3.2. Effect of training set size

**Figure 4:**
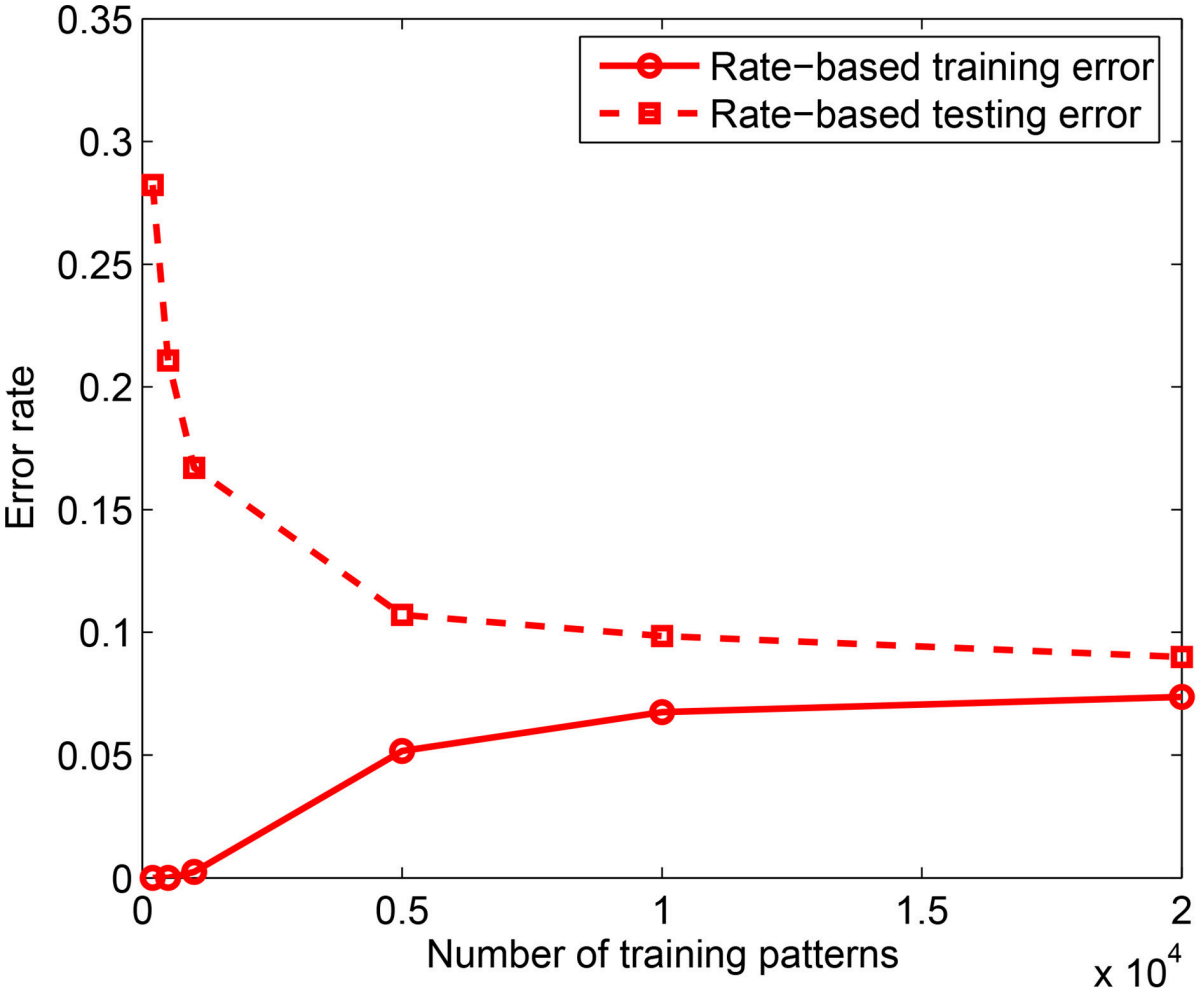
**Training and testing error rates for rate-based learning as a function of the training set size**. Test performance is measured on 10, 000 jittered single spike input patterns with Δ = 10 ms. Each data point is obtained by averaging over 3 training simulation trials and testing on 10 randomly drawn test sets for each learned network; *m* = 10 and *k* = 10.

In the first experiment, we measured the effect of training set size on the classification performance by training the model on *P* = 200 − 20, 000 patterns using the rate-based learning scheme. Each training accuracy is obtained by averaging over 3 trials and the testing accuracy is computed by averaging across 10 presentations of 10, 000 test patterns for each of the learned network, where testing is done on jittered single spike inputs with Δ = 10 ms. The training was done on a network consisting of *m* = 10 dendrites in the PDT and NDT of all *N*_*C*_ = 10 classes. Hence, the total number of dendrites used is *M* = 200. As shown in Figure 4, for the rate-based learning, the training error increases while the testing error on spike versions of the inputs reduces as the training set size increases. It is clear from this result that as more data is added, it becomes difficult to memorize the data. However, the generalization performance improves with more training data. A comparison between the spike-based and rate-based learning schemes is included in the Appendix, where we have shown that we can achieve similar performance for these two forms of learning. However, due to the long simulation times of the spike-based learning, we have used rate-based learning for the remaining analyses in the paper.

### 3.3. Performance of the ensemble method

**Figure 5:**
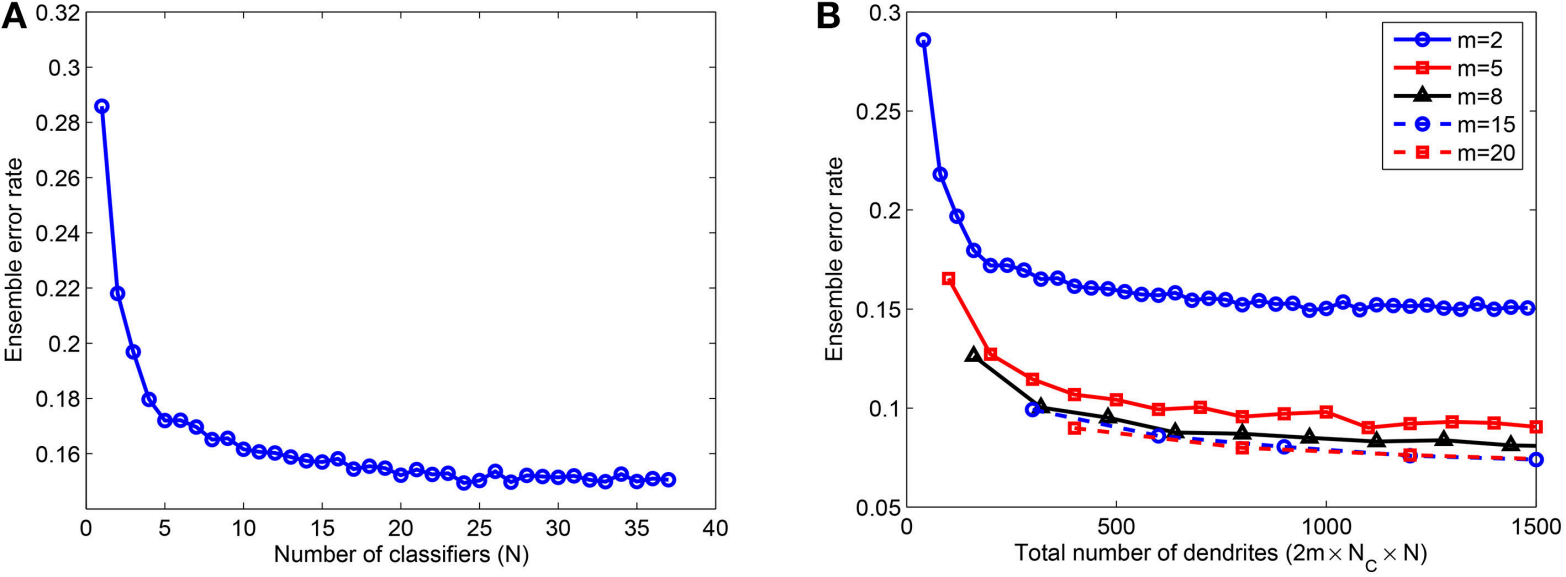
**(A)** Ensemble error rate as a function of the number of classifiers combined, *N*, for 10, 000 test samples; *m* = 2 and *k* = 10. **(B)** Ensemble error rate as a function of the total number of dendrites used, *M*, showing that a moderate number of dendrites per dendritic tree (*m* = 8) gives reasonably good performance and there is no significant improvement when *m* is further increased. Training done on *P* = 5000 patterns randomly drawn from the MNIST samples. Each data point is obtained by randomly selecting a subset (*N*) of the total number of learned classifiers for each case of *m* (37 for *m* = 2). This process is repeated 10 times and the average of the test errors of the combined classifiers is computed.

Next, we studied the effect of combining several NLD multiclass classifiers in an ensemble. These individual classifiers were trained on *P* = 5000 patterns using rate-based learning rule and tested on 10, 000 jittered single spike inputs with Δ = 10 ms. The network consisted of *m* = 2, 5, 8, 15, and 20 dendrites in PDT and NDT for each of *N*_*C*_ = 10 classes. Hence, the total number of dendrites used in the ensemble is given by *M* = 2 × *m*×*N*_*C*_ × *N*. As shown in Figure 5A, the error rate for *m* = 2 reduces by about 48% when up to 25 classifiers are added in the ensemble. However, the error rate doesn't change by much or it increases slightly if further classifiers are combined. Moreover, most of the error reduction, 40% out of the total change of 48%, is achieved when first 5 classifiers are added. We have also looked at the effect of size of individual networks in the ensemble. Figure 5B illustrates this effect where error rate is plotted as a function of the total number of dendrites in the ensemble, *M*. As shown, the error rate reduces with the number of classifiers for all values of *m*. For a fixed value of *M*, *m* = 2 gives the highest error rate, which reduces with larger values of *m*. However, as *m* is increased beyond a certain value (*m* = 8 in this case, the reduction in errors is not significant, and therefore the use of more complex dendritic trees is not leading to significant advantage in terms of performance.

These results indicate that the ensemble model can attain reasonably good performance when the member classifiers are trained using a moderate number of dendrites. In an ideal scenario, we can use a large number of classifiers to achieve significant improvement in performance. However, it is impractical to train several classifiers and hence, we use only a few classifiers which result in most of the performance gain of the ensemble. In case of limited resources (total number of dendrites *M*), an intermediate level of complexity of the ensemble comprising a moderate number of member classifiers with a moderate number of dendrites in each classifier can be used. Next, we investigate how to determine this intermediate level of complexity for a particular classification problem. For an ensemble created with a few classifiers (3 − 5), we do not know the number of dendrites required by each classifier and also further, if different levels of network complexity underlie representation of different classes to which data belongs. Therefore, we use the adaptive learning rule to automatically learn the number of dendrites suitable for each class of a given classification problem.

### 3.4. Performance of the adaptive learning scheme

**Figure 6:**
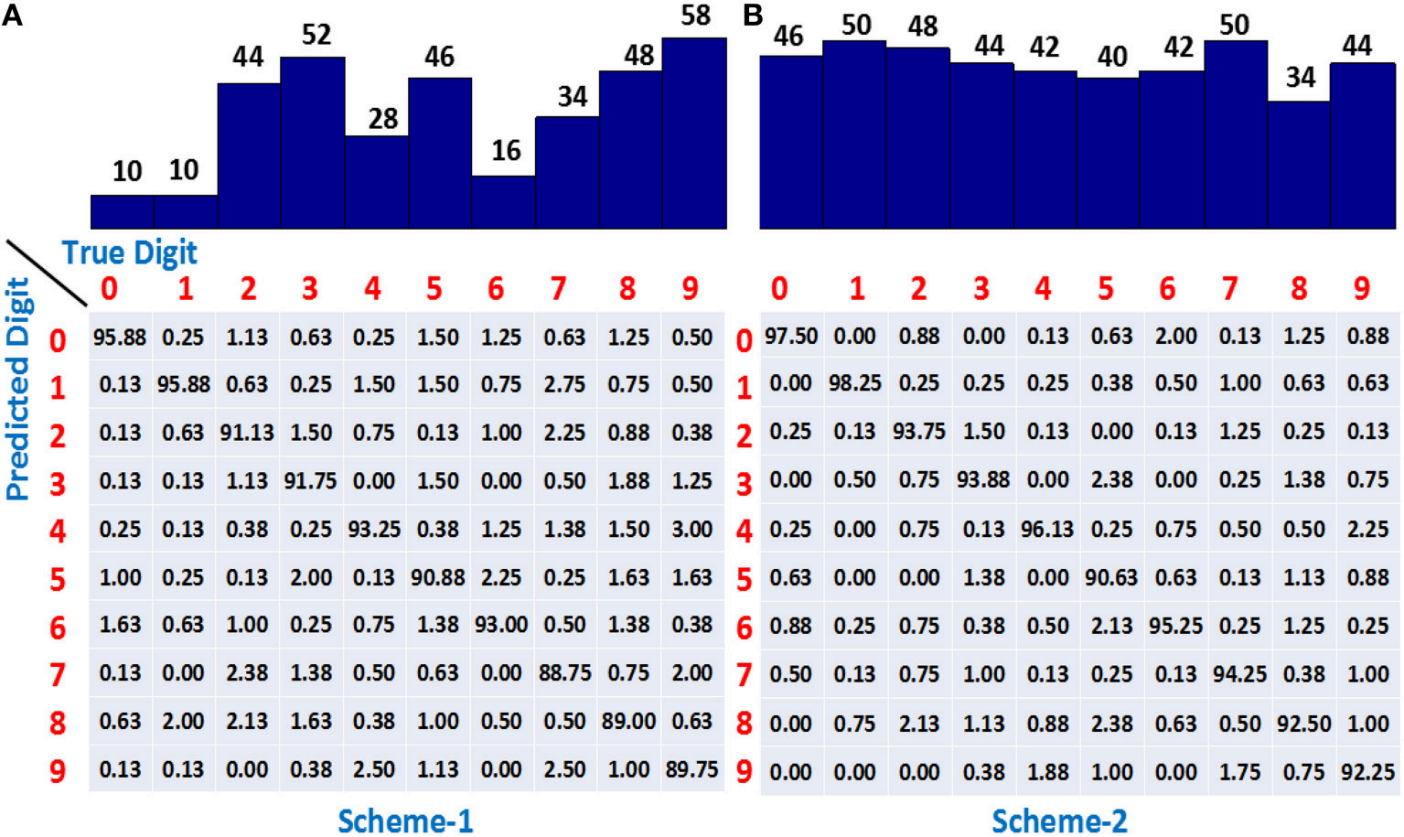
**(A)** The number of dendrites learned using adaptive scheme-1 (top) and the resulting confusion matrix (bottom) showing higher accuracy for easier classes (0, 1, 6) and lower accuracy for difficult digits (8, 9). **(B)** Number of dendrites learned using adaptive scheme-2 (top) demonstrating all the class neurons adding dendrites when required. Confusion matrix (bottom) showing improved accuracy for all the classes. Training set consists of 20, 000 binary digit samples and testing done on 10, 000 jittered single spikes (Δ = 10 ms). Model was initialized with *m* = 5 dendrites for all the classes.

We have used the adaptive learning scheme in which *P* = 20, 000 patterns were used for training by initializing the network with *m* = 5 dendrites in each dendritic tree of a class, and then adaptively increasing *m* in a class-specific manner. The learning process was stopped when 150 minima were encountered and therefore, each adaptively learned classifier took different number of iterations to complete 150 minima. If dendrites are added to only 5 worst-performing classes whenever their learning slows down, referred to as scheme-1, the accuracy obtained on 10, 000 test single spike inputs is 92.1%. The number of dendrites learned by each class in one simulation run is shown by the bar plot at the top of Figure 6A. It can be seen that the digits “2,” “3,” “5,” “8,” and “9” use most of the dendrites and hence are most difficult to learn while “0,” “1,” and “6” are the easier ones requiring only a small number of dendrites. The confusion matrix at the bottom of Figure 6A shows the classification accuracy when an actual digit (column-wise) is predicted by the model as represented row-wise. We can see that the neurons for easier digits “0,” “1,” and “6” can attain good accuracy by utilizing small number of dendrites whereas the difficult digits like “8” and “9” exhibit lower accuracy, where “9” is mostly misclassified as “4” and “7” having similar features.

A drawback of scheme-1 is that we are not allocating the resources appropriately in this case since adding dendrites to only “difficult to learn” classes might not be helpful beyond a point and adding dendrites to the easier classes also can allow the learning to converge faster and attain better performance. This idea was supported by our results obtained by adding dendrites to all the classes, referred to as scheme-2, which yielded a classification accuracy of 94.2%. The number of dendrites learned by all classes is shown at the top of Figure 6B, indicating that the dendrites are added to not only the difficult classes but the easier digits like “0,” “1,” and “6” also utilize more resources. The confusion matrix shows that the classification accuracy of almost all the digits increase as compared to scheme-1, thereby contributing to the overall improved performance.

### 3.5. Test of noise sensitivity

**Figure 7:**
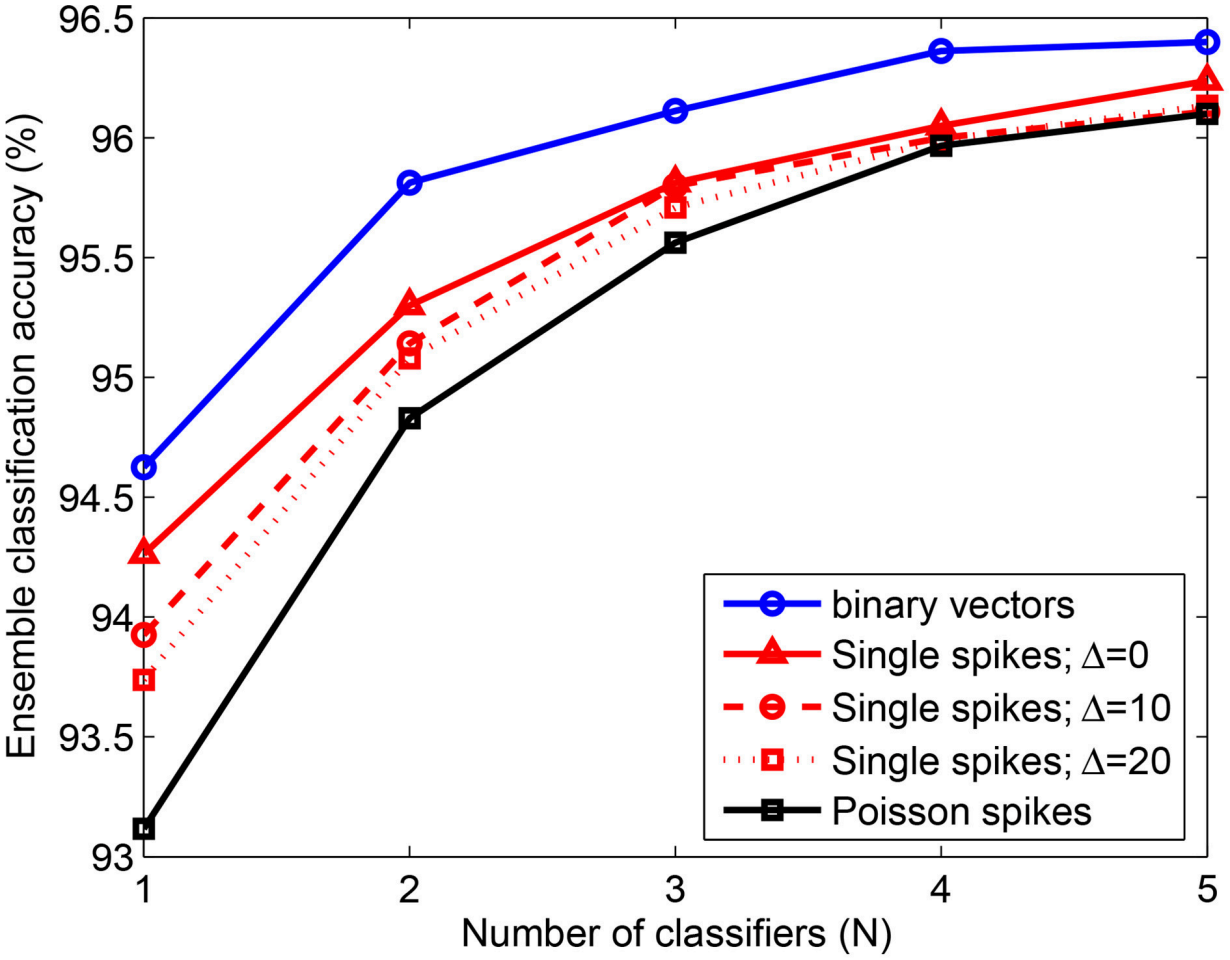
**Error rate of ensemble consisting of NLD classifiers trained on *P* = 20, 000 MNIST samples using the adaptive scheme-2**. The ensemble accuracy on 10, 000 test patterns is computed for binary vectors and spike inputs with different levels of noise. The accuracy obtained for single spike inputs with time window size Δ = 20 ms is 96.1% by combining 5 classifiers. For Poisson spike inputs, *f*_*high*_ = 250 Hz and *f*_*low*_ = 1 Hz. Each data point is obtained by randomly selecting a subset (*N*) of the total number of learned classifiers (5). This process is repeated 5 times and the average of the test errors of the combined classifiers is computed.

We have also tested the performance of an ensemble created with classifiers learned using the adaptive scheme-2, in the presence of noisy spike inputs. Figure 7 shows the classification accuracy for 10, 000 test patterns consisting of single spike inputs with different levels of jitter Δ and Poisson spike inputs with *f*_*high*_ = 250 Hz and *f*_*low*_ = 1 Hz. For reference, we also present the testing accuracy obtained when non-spiking binary inputs are used (solid blue), which increases from 94.6% to 96.1% by adding just 3 classifiers to the ensemble and further increases to about 96.4% by adding 2 more classifiers. The accuracy for single spikes with no noise (Δ = 0) is about 0.2 − 0.5% less than that of binary vectors, which can be attributed to the effects of integrate and fire process of the neurons in the model. As noisy single spikes are presented, accuracy reduces further. However, the change is only about 0.5% for Δ = 20 ms as compared to Δ = 0. Further, the performance of the ensemble on spiking inputs with and without noise become very similar when just 3 dendritic classifiers are combined together. Similarly, for Poisson spike inputs the ensemble yields similar classification accuracy as in the absence of noise. Hence, the ensemble consisting of a few classifiers where each classifier is individually trained using the adaptive learning scheme offers the following advantages: (1) performance gain; (2) feasible to train few classifiers; (3) automatically learned network complexity; (4) noise sensitivity and (5) limited resources due to the use of a few classifiers with adaptively learned network size.

### 3.6. Analysis of dendrite weights: feature maps

We also analyzed the weights of dendrites to understand the features of inputs learned by the nonlinear dendrites. Since our learning rule encourages correlated inputs to be grouped together on the same dendrite, we expected the dendrite weight maps to capture the input correlations. For this purpose, we mapped the *d* = 784-dimensional binary weight vector *w*_.*j*_ = [*w*_1*j*_*w*_2*j*_ … *w*_*dj*_] for the *j*th dendrite to a 28 × 28 matrix consisting of binary elements (since there can be multiple connections from the same afferent on a dendrite, the weight vector can have integer values >1). For this analysis, 20, 000 patterns were trained using the adaptive scheme-2 by initializing the network with *m* = 5 dendrites for each class.

**Figure 8:**
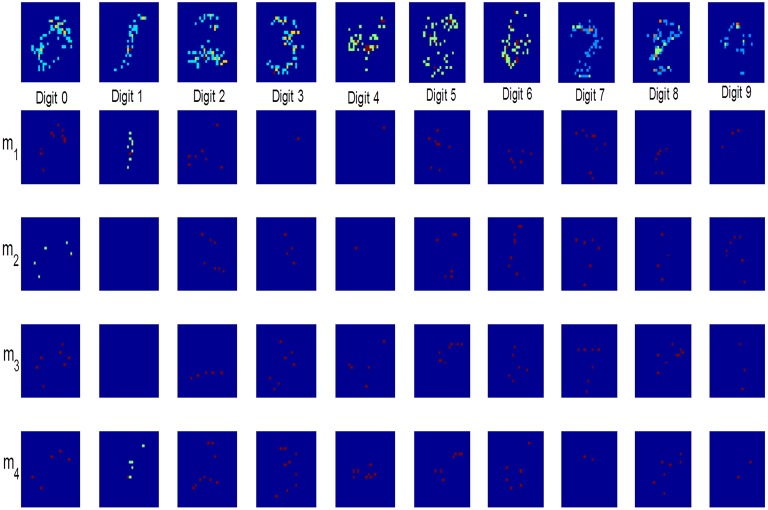
**Weight maps of all dendrites of neurons belonging to a particular digit class (top row)**. Maps in the remaining rows show individual dendrite weights with completely or partially learned features of input digits.

The weight matrices are visualized as shown in Figure 8. The first row depicts the mean weights of all the dendrites corresponding to a digit. It can be seen that the dendrites learn the features of digit images presented to the network. The dendrites belonging to a particular digit class together represent the complete digits. The feature maps look pixelated due to sparse integer weights learned by each dendrite. The remaining rows show the weights of 4 individual dendrites, *m*_1_ to *m*_4_, randomly selected out of the total learned dendrites for each digit. These maps demonstrate that each dendrite learns some features of the input digits. However, some of the dendrites like *m*_4_ of digit “3” and *m*_3_ of digit “7” can learn to represent complete digits, which suggests that some of the dendrites for these classes are redundant and hence, can be removed. A hybrid approach which involves adding new dendrites and pruning the superfluous ones can therefore be used to design the network.

### 3.7. Boosting the performance using optimal neuron topology

**Figure 9:**
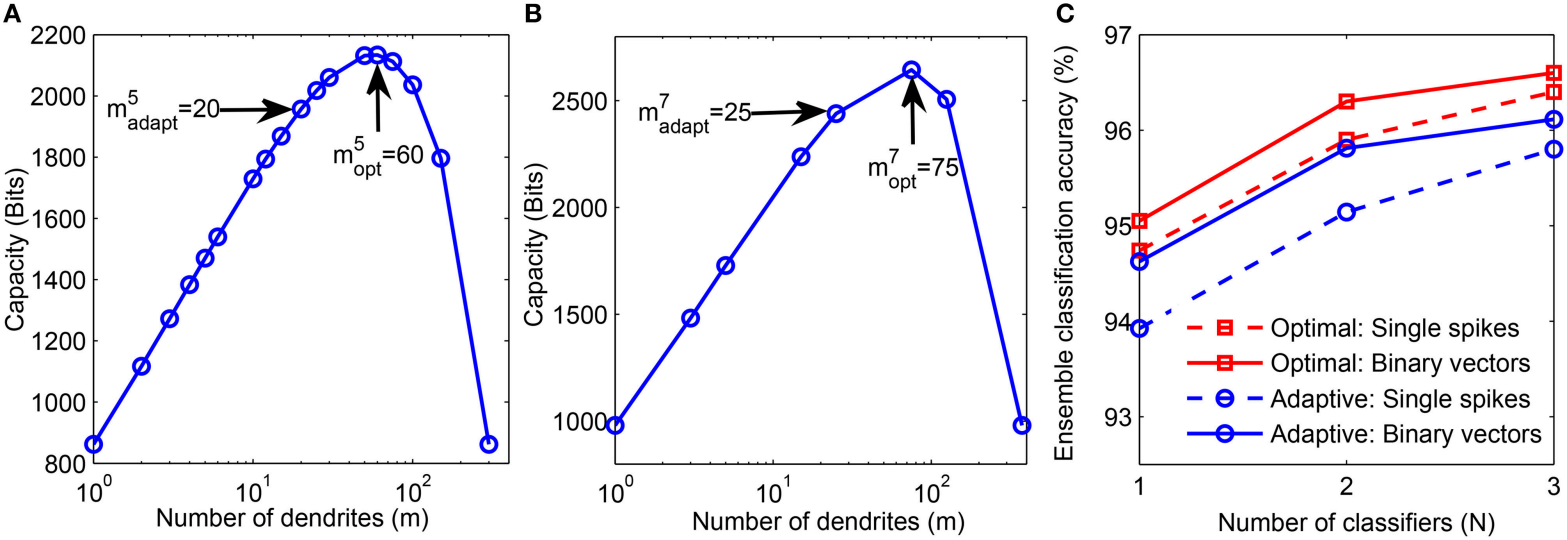
**(A)** Theoretical capacity of neuron corresponding to digit “5” as a function of number of dendrites (*m*). The number of dendrites learned adaptively is $m_{adapt}^{5}$ and the optimal number of dendrites corresponding to maximum capacity is $m_{opt}^{5}$. **(B)** Adaptively learned and optimal *m* values for digit “7.” **(C)** Performance comparison of adaptively learned network (blue) with theoretically determined optimal network (red). Training was done on 20, 000 binary digit samples and test performance was measured on 10, 000 binary inputs (solid) and jittered single spike input patterns with Δ = 10 ms (dashed).

Next, we determine the theoretically optimal configuration of the network which can be used to boost the performance of our structural learning scheme, as discussed in Section 2.1.3. For this analysis, we used one instance of the final number of dendrites learned for each class using the adaptive scheme-2 (Figure 6B). We then computed the theoretical capacity for each neuron of all classes by fixing the total number of synapses learned by the μ^*th*^ neuron, $s_{adapt}^{\mu }$, and varying the *m* and *k* values such that $s_{adapt}^{\mu }=s_{opt}^{\mu }=m\times k$ (step-2 in Figure 3). Figure 9A shows the theoretical capacity of class “5” neuron plotted as a function of *m*, where $m_{adapt}^{5}$ is the number of dendrites learned using the proposed adaptive method while $m_{opt}^{5}$ is the number of dendrites corresponding to the maximum capacity for a neuron with the same $s_{opt}^{5}=s_{adapt}^{5}$ as that of a neuron trained adaptively. The number of dendrites, $m_{adapt}^{7}$ learned by our method and the corresponding optimal value, $m_{opt}^{7}$ for class “7” neuron are shown in Figure 9B.

We then trained a network with neurons consisting of $m_{opt}^{\mu }$ dendrites on 20, 000 MNIST samples using our structural learning rule. Figure 9C shows the comparison between the performance of our adaptively learned network (blue) and the corresponding theoretically optimal network (red). These classifiers were combined in ensembles and tested on 10, 000 non-spiking binary inputs and jittered single spike patterns. The results show that the optimal topology can achieve about 0.5 − 0.9% higher accuracy than the adaptively learned configuration. Moreover, the use of optimal *m* and *k* values also helps to generate the highest accuracy of our model, 96.6%, obtained on the binary test inputs. Further advantage of using a network with optimal topology can be understood by comparing these results with the accuracies depicted in Figure 7. We can see that the ensemble of 3 classifiers with optimal network topology can attain higher accuracy than the ensemble of 5 adaptively learned classifiers. Hence, theoretical capacity predictions of the optimal network configuration can be used to boost the classification performance of our structural learning rule while using same synaptic resources as the adaptive case.

### 3.8. Classification of event-based MNIST-DVS dataset

We also evaluate the performance of our adaptive learning algorithm on the actual event-based MNIST dataset consisting of dynamic vision sensor (DVS) recordings of different handwritten digits (Serrano-Gotarredona and Linares-Barranco, 2014). This dataset was generated by using 10, 000 of 28 × 28 pixel digit images from the original MNIST dataset which were enlarged to three different scales using smoothing interpolation algorithms. These upscaled digit images were displayed on an LCD monitor with slow motion and a 128 × 128 pixel AER DVS (Serrano-Gotarredona and Linares-Barranco, 2013) was used to record these moving digits for a total time duration of 2 s. We used 10, 000 recordings of moving digits upscaled to scale-4 for our analysis, which were also used to evaluate the performance of the event-driven categorization system proposed in Zhao et al. (2015). The training was performed on randomly selected 90% of the total 10, 000 recordings while the remaining 10% recordings were used for testing. For training, the event streams for 128 × 128 DVS recordings were converted to 128 × 128 pixel images by calculating the total number of events occurring at each pixel location for two different time durations, 100 ms and full length of 2 s. The 128 × 128 images were then cropped to digit patches by selecting the location of 28 × 28 squares with maximum events occurring.

The rate-based adaptive scheme was used for training followed by testing on event streams occurring at the 28 × 28 patch extracted from the original 128 × 128 DVS recordings. The training procedure was repeated 3 times and the average testing accuracy over 3 trials was determined as shown in Table 3, which also compares with the performance of the event driven system in Zhao et al. (2015). The results show that our adaptive dendritic algorithm can achieve comparable testing accuracy (88.1% for 2 s) with the other reported performance on MNIST-DVS dataset, with higher accuracy attained when longer recordings are used. Moreover, our method yields lower training accuracy than Zhao et al. (2015) while the testing accuracy is similar, suggesting that our method is more robust to overfitting.

**Table 3:** **Performance on MNIST-DVS dataset**.

**Time length of recording used**	**Training accuracy (%)**	**Testing accuracy (%)**
100 ms (Zhao et al., 2015)	98.9	76.9
2 s (Zhao et al., 2015)	99.1	88.2
100 ms (this work)	93.7	80.2
2 s (this work)	97.3	88.1

### 3.9. Comparison with other spike-based classification models

Finally, we compare the performance of our model with other spike classification models consisting of a network of spiking neurons. The spike classifiers considered here use the two main approaches of rate-based and spike-based learning. The models consisting of spiking Restricted Boltzmann Machines (RBM) map the weights learned using offline rate-based scheme onto spiking neural network in O'Connor et al. (2013) and use a spike-based event-driven learning rule based on STDP in Neftci et al. (2014). In Brader et al. (2007), a stochastic spike-driven synaptic plasticity rule was used to train a network of binary synapses and classification accuracy determined by voting over a pool of neurons. Table 4 compares the training, test sizes, number of neurons or dendrites, synapses used and the accuracy attained by these classification algorithms on MNIST dataset. The number of neurons mentioned is the total number of neurons in the network excluding those in the input layer. For our model, each dendrite can be considered as a processing subunit and is therefore also shown here. For the other networks, all synaptic currents sum up linearly implying the use of only one dendritic branch per neuron. The number of training examples used by Brader et al. (2007) and Neftci et al. (2014) and our method is 20, 000 while the training set in O'Connor et al. (2013) consisted of 120, 000 samples generated by introducing small random translations, rotations and scalings in the original MNIST training examples. The number of test patterns used by all the models is 10, 000.

**Table 4:** **Comparison with spike classifiers on MNIST data**.

**Model**	**#Train**	**#Test**	**#Neurons**	**#Dendrites**	**#Synapses**	**Accuracy %**
O'Connor et al., 2013	120, 000	10, 000	1010	1010	647, 0000	94.09
Neftci et al., 2014	20, 000	10, 000	540	540	412, 000	91.9
Brader et al., 2007	20, 000	10, 000	150	150	117, 600	096.5
Adaptive NLD	20, 000	10, 000	20	440	6720	094.2
Ensemble NLD	20, 000	10, 000	100	2312	35, 285	096.1
Optimal NLD	20, 000	10, 000	100	3960	20, 163	96.4

The results suggest that the NLD model can achieve accuracy comparable with other algorithms by utilizing significantly less, which is about 2 − 7% of the total number of synapses used by the other methods. Moreover, the use of low resolution integer weights in our work in contrast with the high resolution weights used by spiking RBM models (O'Connor et al., 2013; Neftci et al., 2014) renders our structural learning rule for NLDs more feasible for implementing spike classification in hardware. Further, the ensemble of NLD classifiers yielded 96.1% accuracy by using rate-based learning and testing on Poisson spike inputs. Moreover, the use of optimal network topology, which was determined using the theoretical capacity calculations, enabled us to attain even higher accuracy by reducing the synaptic resources by about 1.7 times. These results are at par with the best performance achieved by Brader et al. (2007). It is important to note that both models in Brader et al. (2007) and our work use binary synapses; however, we obtain similar performance as in their work by training an ensemble of only a few classifiers resulting in still less number of synaptic resources, about 20% of that used in Brader et al. (2007). We also expect that our learning rule will be more amenable for hardware implementation since the dendritic polynomial nonlinearity is much simpler than implementation of a full neuron as in the population of output neurons in Brader et al. (2007). Therefore, the use of much less number of synaptic resources with binary weights and a more simpler learning rule render our NLD model more hardware-friendly.

## 4. Discussion

Here, we discuss the neurobiological relevance of our work and its potential for future hardware implementation. We also compare our method with other studies based on these neurobiological mechanisms. Finally, we discuss the future directions of our work.

### 4.1. Role of dendritic nonlinearity in neuronal processing

Several experimental evidences support the nonlinear processing in dendrites including active backpropagation of axonal spikes into the dendritic tree and dendritic spikes (Hausser et al., 2000; Schiller et al., 2000). However, there are not many evidences regarding the role of these nonlinear mechanisms in synaptic integration in pyramidal neurons. Experimental and compartmental modeling studies of pyramidal neurons have indicated that nearby synaptic inputs on the same dendrite sum sigmoidally while inputs on different dendrites sum up linearly (Poirazi et al., 2003b; Polsky et al., 2004). These findings support the notion of a two-layer model of neurons, thereby having implications for the synaptic plasticity and the computational capacity of cortical tissue. Mel and his group presented several computational studies to elucidate the role of dendrites in neuronal processing. (Mel, 1991; Poirazi et al., 2003b). In more recent studies, an abstract two-layer model using sigmoidal dendritic nonlinearity was shown to predict the firing rate of a detailed compartmental model of a pyramidal neuron (Poirazi et al., 2003a) and much larger storage capacities were computed for dendritic neurons with degree 10 polynomial nonlinearity in Poirazi and Mel (2001). In contrast to these studies, we use a more hardware-friendly quadratic nonlinearity which is easier to implement than a sigmoid or a high order polynomial. We also modify the learning rule to adapt the structure of the dendritic tree of different neurons in the network according to difficulty of the classification task.

### 4.2. Structural plasticity as a learning mechanism

The phenomenon of structural plasticity involving formation and elimination of synapses thereby leading to alterations to the cortical wiring diagram (Chklovskii et al., 2004; Butz et al., 2009) provides for alternative form of long term information storage in addition to the traditional synaptic weight plasticity. The information storage capacity associated with structural plasticity lies in the ability to change wiring diagram in a sparsely connected network, which provides a large number of functionally distinct circuits available to encode information (Chklovskii et al., 2004) and hence has important implications for the computational properties of the network. The computational modeling study by Poirazi and Mel (2001) demonstrated the use of structural plasticity to modify binary synaptic connections on dendritic branches. Similar to our model, a poorly performing active synapse is eliminated and replaced by the best performing synapse in a set of silent synapses. However, our learning rule is simple and easier to implement in hardware systems as compared with the learning rule used by Poirazi and Mel (2001).

### 4.3. Binary synapses: computational challenges

There is accumulating experimental evidence that biological synapses exist in only a small number of states which can be restricted to even two states (Petersen et al., 1998; O'Connor et al., 2005). The use of synapses with only one or two bits of long-term information has severe implications for the storage capacity of networks working as classifiers or associative memories with capacity for binary synapses reducing by more than half as compared to the capacity using continuous-valued synapses (Senn and Fusi, 2005). Some studies have presented learning algorithms as biological solutions to deal with the reduced storage capacity of networks with binary synapses. A stochastic spike-driven synaptic plasticity rule was used to train a network of binary synapses, where a pool of output neurons was used to calculate the classification accuracy by a voting mechanism (Brader et al., 2007). This results in a large number of synapses being used. In comparison to this study, our model employs a sparsely connected network of binary synapses which learns by using a correlation-based structural plasticity rule. The use of dendritic nonlinearity yields higher computational power thereby alleviating the problem of reduced capacity of binary weights. Also, the adaptive learning of number of dendrites according to problem complexity reduces the number of synapses compared to a brute force approach. Hence, our model can achieve higher accuracy by utilizing a small number of binary synapses.

### 4.4. Binary synapses and structural plasticity: considerations for hardware implementation

Over the past decade, several low-power neuromorphic systems have been built to perform classification of spike patterns. A common feature in several of these systems is the usage of binary synapses (Indiveri et al., 2006; Arthur and Boahen, 2007; Mitra et al., 2009). One reason for this is the ease with which two states can be stored in current CMOS technology using a latch. This also makes the system more robust to parametric variations due to mismatch in device - it is unlikely that high resolution weights can be obtained from a massive array of analog synapses due to a combination of systematic and random mismatch (Linares-Barranco et al., 2003). Even a recently introduced multi-core asynchronous digital chip (Merolla et al., 2014) uses a limited number of weight values per axon per core. Our algorithm is consistent with this philosophy of low-resolution weights since we limit the number of synaptic connections per dendrite and each connection is a binary value. Effectively each input afferent (or axon) connects with a small integer weight to a dendrite.

Another advantage of our architecture is that the learning happens by modifying connectivity patterns of the network. In most current event-based neuromorphic systems, this connection matrix is stored in a separate memory (Liu, 2014) either on or off chip. This implies that since our hardware architecture enforces sparsity, we require less memory and memory reads to store and access connection information respectively. Before expanding on this point, it is important to note that we are not considering advantages of hardware implementations of on-chip learning to find optimal connections (though we have presented some initial results on the same in Roy et al., 2014b). We are only comparing the advantages of using our proposed architecture to implement the final network and using structural plasticity to reduce the memory requirement of this implementation. In this context, it should be noted that normal weight learning methods do not necessarily produce sparse weights and simple quantization of small weights to zero values increase errors. This was shown to be true for an ensemble of perceptrons trained by the p-delta algorithm in Roy et al. (2014a). More recently, there have been efforts to improve rounding algorithms to reduce weight resolution for efficient implementation of deep networks (Muller and Indiveri, 2015). Even with these methods, a two layer fully connected neural network with 500 hidden nodes needs at least 4 bits per synaptic weight to achieve comparable performance (~96%) as our network on the MNIST dataset. This results in approximately 397, 000 4-bit weights as opposed to ~21, 000 1-bit weights in our case.

To generalize this result, let us consider a two layer network for the conventional case with *d* inputs, *H* hidden layer neurons and *C* output neurons for “C” classes. The comparable network in our proposed case has *H* dendrites and *C* output neurons. “2m” out of the *H* dendrites connect to each of the *C* output neurons (*H* = 2*m* × *C*) using unit weights and hence can be implicitly implemented by accumulators. Considering each weight of the conventional network having resolution of “b” bits, the total number of bits required by the conventional network (*NOB*_*conv*_) is given by:

(22) $$NOB_{conv}=b\times H\times d+b\times H\times C\approx b\times H\times d\text{for}C<<d$$

**Figure 10:**
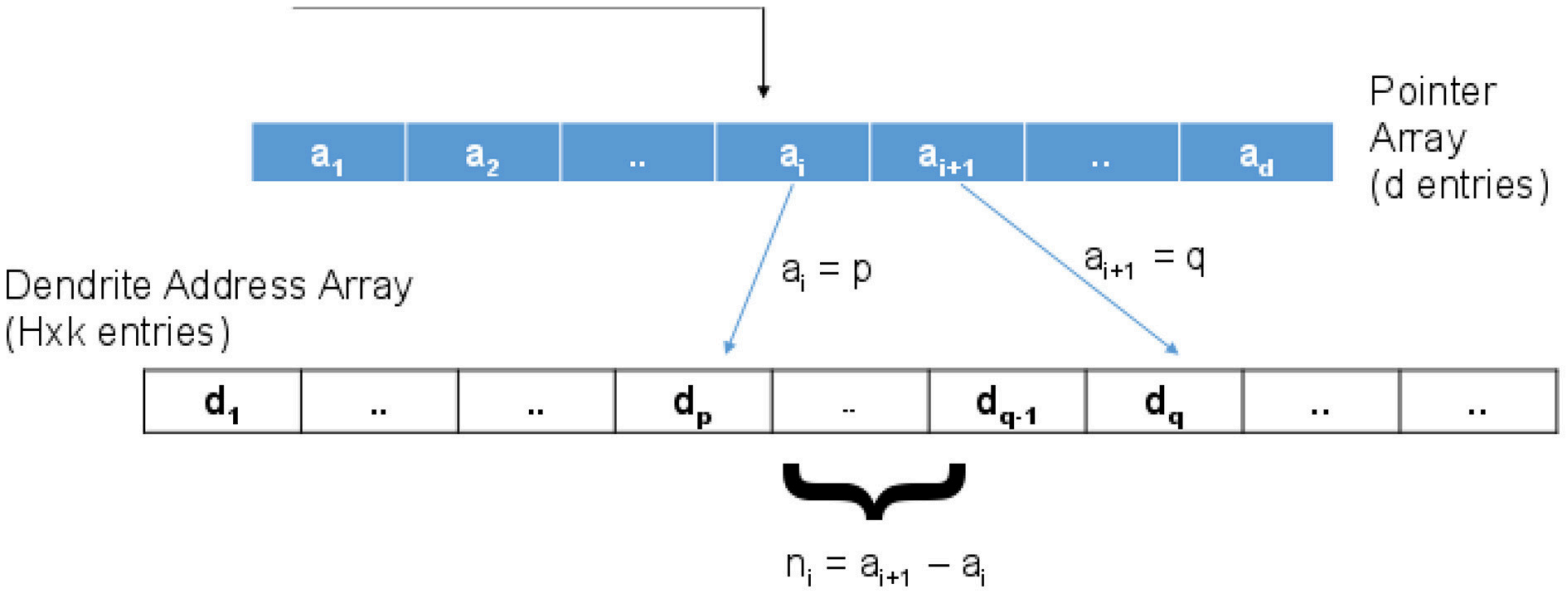
**To implement the sparse connectivity matrix, a two level addressing scheme is proposed where the first memory (indexed by the incoming address of the event) holds pointers to valid connection addresses stored in the second memory**.

For the proposed case, the connection matrix is of size *d* × *H* though only *k* × *H* entries are non-zero where *k* < < *H*. To implement this sparse connectivity efficiently in an address event framework, we propose to use a two tier addressing scheme as shown in Figure 10. Here, the incoming address will be used to index into a pointer array of “d” entries with ⌈*log*_2_(*H* × *k*)⌉ bits per entry. An incoming spike address, say “i,” is used to index into this array and read the two consecutive values *a*_*i*_ and *a*_*i* + 1_. As shown in the figure, suppose *a*_*i*_ = *p* and *a*_*i* + 1_ = *q*. *n*_*i*_ = *a*_*i* + 1_ − *a*_*i*_ is the number of synapses connected to this input. If *n*_*i*_ > 0, then *a*_*i*_ = *p* is used as a pointer to the *p*th location in a dendrite address array. This second array has *H* × *k* entries with ⌈*log*_2_(*H*)⌉ bits per entry that hold the address of the dendritic branch where the synapse is located. *n*_*i*_ consecutive values (*d*_*p*_ to *d*_*q* − 1_) are read as destination addresses to route the spikes. Now, the total memory required by the look up table in the proposed method (*NOB*_*prop*_) can be estimated as:

(23) $$NOB_{prop}=d\times \lceil log_{2}(H\times k)\rceil +H\times k\times \lceil log_{2}(H)\rceil$$

**Figure 11:**
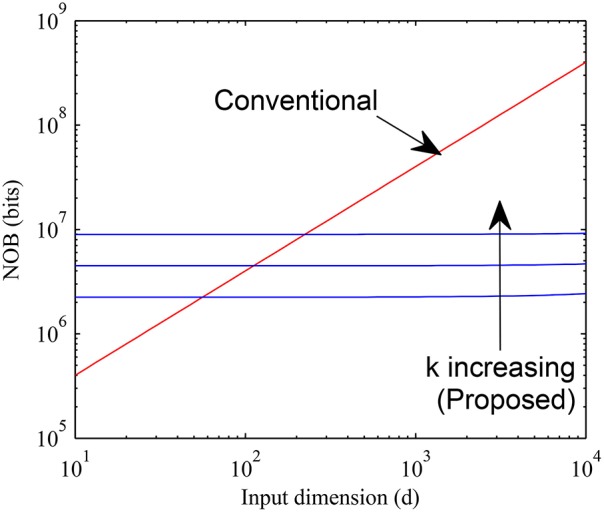
**Memory requirement of proposed sparse connectivity scheme is compared with that of a normal fully connected weight memory with 4 bits per weight**. The proposed scheme is far more memory efficient for high dimensional inputs (*d* > 200).

The memory requirements for proposed and conventional methods are compared in Figure 11 by setting *b* = 4, *H* = 10^4^ and varying d over a wide range for *k* = 16, 32, and 64. It can be seen that the proposed method requires much less memory than the conventional case for large values of *d* when the sparsity is higher while the overhead of having a pointer array is more for small values of *d*. The crossover typically happens for *d* < 200 for values of *k* as large as 64. Since for most practical cases *d* is much larger, we expect our method to be widely applicable.

To underline the importance of this memory reduction, we consider a digital implementation of this system following principles similar to the ones in Merolla et al. (2014) and Seo et al. (2011). In particular, we assume that the dendritic nonlinearity/hidden neuron can be a shared physical circuit that can be time multiplexed across all required instances and we assume synaptic weight resolution is 4 bits following Seo et al. (2011). Here, for simplicity we ignore the overhead needed if the network is spread across multiple cores. Using the numbers quoted in Merolla et al. (2014), the area requirement of a neuron circuit is 2900 μ*m*^2^. Compared to that, the area required to implement a conventional crossbar of 4 bit weights for the MNIST case of *d* = 784 and say *H* = 1000 is 470, 400 μ*m*^2^ where we estimate 0.15 μ*m*^2^ area per bit from Merolla et al. (2014). This is clearly the dominant factor in chip area. Compared to this, our scheme with even *k* = 64, *H* = 1000 and *d* = 784 requires approximately 98, 000 μ*m*^2^ area, a reduction by > 4*X*.

### 4.5. Future work

The classification performance attained by our model on the benchmark MNIST data is not state-of-the-art. The best MNIST classification result achieved so far is 99.06% accuracy using maxout networks (Goodfellow et al., 2013). Hence, we need to bridge this gap by enhancing our model. Our present model consists of lumped dendritic nonlinearity such that each dendrite is considered to be a single compartment where all the synaptic inputs are lumped together. The storage capacity of this network can be increased by introducing multiple compartments on each dendrite. The dendritic compartments represent time delays in signal propagation along a dendrite and therefore, the information about the location of synaptic inputs on a dendrite is important. We will utilize this additional source of spatial information to enhance our dendritic structural learning rule which will involve finding the optimal location on the optimal dendritic branch for a synaptic connection.

The storage capacity of the network can further be increased by including distributed nonlinearity along a dendrite such that the nonlinear output of each compartment serves as input to the next compartment on the dendrite. This scheme is also more bio-realistic from the perspective of real neurons consisting of extended dendritic trees with complex branching patterns. We will also enhance our adaptive learning rule to prune the redundant or least “salient” synapses. This pruning method combined with the progressive addition of dendrites will yield an optimally sized network that will fit the data. The network will learn both the number of required dendrites as well as the number of synapses on each dendrite. This approach to obtain the smallest network can also improve the generalization performance.

In this work, we have not used temporal information to classify input patterns and have focussed on rate and place encoding of the binary images from the MNIST dataset. In a recent work (Roy et al., 2015), we have used structural plasticity to learn binary classification spatiotemporal patterns as used in Gutig and Sompolinsky (2006). Hence, a natural extension of our present work is to combine the use of spike timing information with structural learning to enable classification of multiclass temporal codes.

## Author contributions

SH and AB developed the idea for the paper. SH did all the modeling simulations. SH analyzed the results and both authors discussed the results and contributed to the manuscript preparation. AB supervised this work. This work was supported by MOE, Singapore through grant ARC 8/13.

### Conflict of interest statement

The authors declare that the research was conducted in the absence of any commercial or financial relationships that could be construed as a potential conflict of interest.

## Figures and Tables

**Figure A1:**
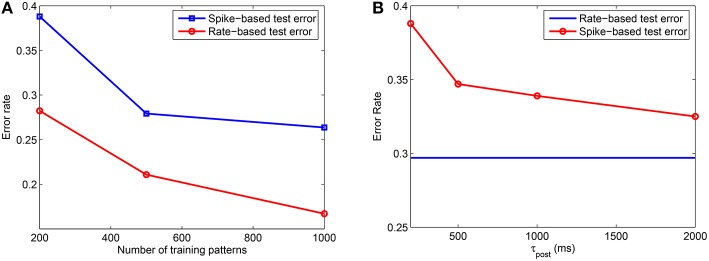
**(A)** Comparison of performance of rate-based and spike-time-based learning schemes. The test error rates for the spike-based learning rule are about 1.5 times higher than the rate-based test errors; **(B)** Comparison of test error rate for *P* = 200 patterns learned with rate-based (blue line) and spike-based (red plot) methods as a function of τ_*post*_. Each data point is obtained by averaging over 3 training simulation trials and testing on 10, 000 single spike inputs with jitter Δ = 10 ms; *m* = 10 and *k* = 10.

## A. Appendix

We have proposed a biologically realistic branch-specific spike-time dependent structural plasticity (BSTDSP) rule for binary classification in Hussain et al. (2015), which was inspired from a recent study in which reverse spike-timing dependent plasticity (RSTDP) in concert with hyperpolarization of the postsynaptic neuron was used to modify the synaptic weights (Albers et al., 2013). Our learning rule involving modification of connections instead of synaptic weights, is in contrast with Albers et al. (2013) and is used to find the bio-realistic correlation values *c*_*ij*_. However, the bio-realistic basis of adapting the number of dendrites is not clear.

The correlation values needed for structural plasticity rule were obtained from an online spike-based learning rule. The biological relevance of these correlations can be found in calcium concentration in spines which is a correlation sensitive, spike-time dependent signal and has been implicated as a guide for structural plasticity (Helias et al., 2008). Here, we present an extension of BSTDSP to multiclass classification and also demonstrate the correspondence between rate-based and spike-based learning approaches.

### A. Spike-based structural learning for multiclass classification

The multiclass BSTDSP is derived using the example of synchronous single-spike inputs such that each afferent either fails to fire or fires a spike at a fixed time *T*_*syn*_ and these spike inputs are assigned to *N*_*C*_ classes. The single-spike input is denoted by the presynaptic spike train *s*_*ij*_(*t*) at the *i*th synapse of the *j*th dendrite, and the postsynaptic spike train is denoted by *r*(*t*) given by:

(A1) $$r(t)=\sum_{t_{post}}\delta (t-t_{post})$$

where *t*_*post*_ is the postsynaptic spike time. These pre- and postsynaptic spikes also drive exponentially decaying memory traces–the presynaptic trace $\bar{s}$ and the postsynaptic trace $\bar{r}$ given by:

(A2) $$\bar{s}=exp(-t/\tau_{pre})$$

(A3) $$\bar{r}=exp(-t/\tau_{post})$$

If a pattern belonging to class μ is presented, a teacher signal forcing a postsynaptic spike (at time *t* = 1 ms) is present for the (+) neuron and absent for (−) neuron of class μ. For the remaining (*N*_*C*_ − 1) classes, opposite conditions for the teacher signal exist, i.e., teacher signal is absent for (+) neuron and present for (−) neuron.

The spike-based structural plasticity learning rule is similar to the rate-based learning method and also involves computing correlation values *c*_*ij*_ for each synapse belonging to the PDT and NDT corresponding to class μ. This learning scheme is analogous to the RSTDP rule such that if a postsynaptic spike arrives before a presynaptic spike, then the correlation value for that synapse is potentiated. The condition for depression does not use postsynaptic spike–instead, the relevant event is when the membrane voltage *V*_*m*_(*t*) crosses a subthreshold voltage *V*_*st*_ from below, where 0 < *V*_*st*_ < *V*_*thr*_. These subthreshold crossing events occurring at times *t*_*st*_ are denoted by $r_{st}\left(t\right)=\underset{t_{st}}{\sum }\delta \left(t-t_{st}\right)$. The correlation value is decreased when presynaptic spike time $t_{ij}^{s}$ occurs before *t*_*st*_. The reason for using *r*_*st*_(*t*) instead of *r*(*t*) is to enforce a margin as will be explained later in this section. Finally, the change in correlation value Δ*c*_*ij*_(*t*) for every pattern presentation is computed using these plasticity conditions in a branch-specific manner. Hence, the learning rule for the PDT and NDT belonging to the μth class can be written as:

(A4) $$\begin{array}{l} \text{For PDT of class }\mu :\\ \;\Delta c_{ij}^{PDT}(t)=I_{D,j}^{PDT}(t){\bar{r}}^{PDT}(t)s_{ij}^{PDT}(t)-\\ \;\gamma I_{D,j}^{PDT}(t){\bar{s}}_{ij}^{PDT}(t)r_{st}^{PDT}(t) \end{array}$$

(A5) $$\begin{array}{l} \text{For NDT of class }\mu :\\ \;\Delta c_{ij}^{NDT}(t)=I_{D,j}^{NDT}(t){\bar{r}}^{NDT}(t)s_{ij}^{NDT}(t)-\\ \;\gamma I_{D,j}^{NDT}(t){\bar{s}}_{ij}^{NDT}(t)r_{st}^{NDT}(t) \end{array}$$

where γ is a constant used to balance the potentiation and depression and ensure that *c*_*ij*_ = 0 when a pattern is learned. The correlation values averaged over all the patterns can be written as: $c_{ij}^{PDT}=<\Delta c_{ij}^{PDT}\left(t\right)>$ and $c_{ij}^{NDT}=<\Delta c_{ij}^{NDT}\left(t\right)>$, where < . > indicates average calculated over an epoch. It was shown in Hussain et al. (2015) that by assuming the presynaptic time constant τ_*pre*_ to be much greater than the membrane time constant τ_*V*_, the value of γ can be computed as:

(A6) $$\gamma =\frac{exp(-T_{syn}/\tau_{post})}{exp(-T_{rise}/\tau_{pre})}$$

where the presynaptic spike arrives at time *T*_*syn*_ and *V*_*m*_(*t*) crosses *V*_*st*_ at time *T*_*rise*_. The connection changes are done on the basis of *c*_*ij*_ values using the same process as discussed for rate-based learning. The use of a subthreshold voltage *V*_*st*_ leads to a margin δ_*spike*_ for classifying spike patterns. A desired margin δ_*spike*_ for a particular class μ can also be preset, which can then be used to calculate the class specific values of *V*_*st*_ and *V*_*reset*_ as explained in Hussain et al. (2015). The class margins suited for a particular problem can be assigned by using margins set for rate-based learning. The value of the desired margin, δ_*spike*_ is determined by using the spike equivalent value of δ. This is done by computing the difference in the membrane voltage of the neuron (analogous to *a*_*PDT*_ − *a*_*NDT*_) when a single synapse is activated. This value Δ*V*_*m*_ multiplied by δ gives δ_*spike*_, since in the binary input case the synaptic strength is normalized to 1.

The spike-based and the rate-based structural learning rules were compared in Hussain et al. (2015) and a relationship between the correlation terms [Δ*c_ij_*]_*spike*_ and [Δ*c_ij_*]_*rate*_ for the two forms of learning was derived as given below.

(A7) $${\left[\Delta c_{ij}\right]}_{spike}=exp(-T_{syn}/\tau_{post}){\left[\Delta c_{ij}\right]}_{rate}$$

Therefore, any differences between the performance of the two learning methods can be understood on the basis of this relationship. This suggests that as the value of τ_*post*_ is increased, the correlation values for the rate-based learning, [Δ*c_ij_*]_*rate*_ well approximate the correlation values for the spike-based learning, [Δ*c_ij_*]_*spike*_. Since the two forms of learning tend to coincide with increasing τ_*post*_, therefore, we have used the faster rate-based approach instead of the computationally prohibitive spike-based learning scheme for the enhancements proposed in the model.

### B. Comparison of spike-based learning vs rate-based learning schemes

We have compared the performance of the spike-based and rate-based learning schemes. Since spike-time-based learning takes very long and the training time increases with the size of training set, therefore, we have trained the spike model on smaller number of training patterns, 200 − 1000. For both forms of learning schemes, the networks consisted of *m* = 10 dendrites in the PDT and NDT of all *N*_*C*_ = 10 classes. Hence, the total number of dendrites used by both models is *M* = 200. Figure A1A shows the comparison between the performance of spike-based and rate-based learning schemes. Both forms of learning exhibit the trend of decreasing test error with the training set size. However, the spike-based learning method results in about 1.5 times higher errors than that for rate-based learning. This difference in performance of the two methods can be attributed to three reasons: (1) Firstly, the relationship between these two methods (Equation A7) suggests that as the value of τ_*post*_ is increased, the correlation values for the two learning methods become more similar. Therefore, we need to repeat our spike learning simulations with higher values of τ_*post*_ to achieve greater agreement between the two forms of learning; (2) Secondly, our estimation of γ in Equation (A6) is based on the assumption that τ_*pre*_ ≫ τ_*V*_. Hence, smaller τ_*pre*_ will result in a non-ideal value of γ which will give different results for the two learning schemes; and (3) Finally, the discrepancy in results can arise if the margin setting is not exact. As discussed above, the margin for spike-based learning was set using δ_*spike*_ = Δ*V*_*m*_ × δ, where a wrong estimate of the constant Δ*V*_*m*_ will mean that δ_*spike*_ and δ are not truly analogous to each other, leading to different results.

Based on this discussion, we attempted to reduce the difference between the performance of rate-based and spike-time-based learning approaches. Therefore, we increased the value of τ_*post*_ from 200 to 500, 1000 and 2000 ms and τ_*pre*_ from 10 to 50 ms. As shown in Figure A1B, the test error for τ_*post*_ = 200 ms corresponds to our prior parameter settings for *P* = 200 patterns (Figure A1A, blue curve). As τ_*post*_ is increased, the testing performance of spike-based learning rule improves and gradually becomes closer to the testing performance of rate-based method, shown with blue line. We expect that we can achieve further agreement between the two forms of learning by getting the margin values δ_*spike*_ and δ to be analogous to each other or by determining the desired δ_*spike*_ directly in the spike domain. Hence, we have shown that the error rate for spike-based learning becomes similar to that of rate-based learning if τ_*post*_ is long enough.
